# Magnetization relaxation in the single-ion magnet DySc_2_N@C_80_: quantum tunneling, magnetic dilution, and unconventional temperature dependence†
†Electronic supplementary information (ESI) available. CCDC 1587626 and 1587784. For ESI and crystallographic data in CIF or other electronic format see DOI: 10.1039/c8cp01608a


**DOI:** 10.1039/c8cp01608a

**Published:** 2018-04-10

**Authors:** D. S. Krylov, F. Liu, A. Brandenburg, L. Spree, V. Bon, S. Kaskel, A. U. B. Wolter, B. Büchner, S. M. Avdoshenko, A. A. Popov

**Affiliations:** a Leibniz Institute for Solid State and Materials Research , 01069 Dresden , Germany . Email: f.liu@ifw-dresden.de ; Email: s.avdoshenko@ifw-dresden.de ; Email: a.popov@ifw-dresden.de; b Chair of Inorganic Chemistry I , Faculty of Chemistry and Food Chemistry , Technische Universität Dresden , 01062 Dresden , Germany

## Abstract

Relaxation of magnetization in endohedral metallofullerenes DySc_2_N@C_80_ is studied at different temperatures, in different magnetic fields, and in different molecular arrangements. Magnetization behavior and relaxation are analyzed for powder sample, and for DySc_2_N@C_80_ diluted in non-magnetic fullerene Lu_3_N@C_80_, adsorbed in voids of a metal–organic framework, and dispersed in a polymer. The magnetic field dependence and zero-field relaxation are also studied for single-crystals of DySc_2_N@C_80_ co-crystallized with Ni(ii) octaethylporphyrin, as well as for the single crystal diluted with Lu_3_N@C_80_. Landau–Zener theory is applied to analyze quantum tunneling of magnetization in the crystals. The field dependence of relaxation rates revealed a dramatic dependence of the zero-field tunneling resonance width on the dilution and is explained with the help of an analysis of dipolar field distributions. AC magnetometry is used then to get access to the relaxation of magnetization in a broader temperature range, from 2 to 87 K. Finally, a theoretical framework describing the spin dynamics with dissipation is proposed to study magnetization relaxation phenomena in single molecule magnets.

## Abstract 2

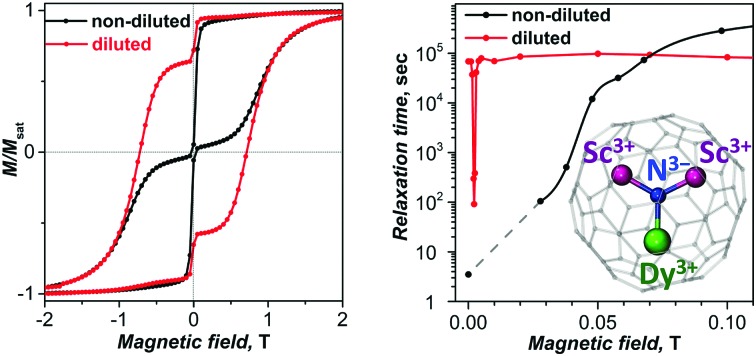
Quantum tunneling and relaxation of magnetization in single molecule magnet DySc_2_N@C_80_ is thoroughly studied as a function of magnetic dilution, temperature, and magnetic field.

## Introduction

Hiding atomic clusters behind the walls of protective carbon cages allows for stabilization of unconventional species, which cannot exist otherwise. When encapsulated clusters comprise metal atoms, such molecules are known as endohedral metallofullerenes (EMFs),^1^–^4^ and when metal atoms are lanthanides, EMFs become magnetic. In so-called clusterfullerenes,^5^ metal atoms are coordinated to negatively charged non-metal units, such as N^3–^, S^2–^, O^2–^, C_2_^2–^, CN^–^*etc.* The presence of such units at a close distance to lanthanides creates strong axial ligand fields (LF, for historical reasons also often termed crystal field, CF) and hence large magnetic anisotropy.^6^–^11^ Additionally, EMFs can contain up to three lanthanide ions within one endohedral cluster, which adds exchange and dipolar interactions on top of single-ion magnetic properties.^6^,^12^–^16^ Large magnetic anisotropy leads to magnetic bistability on a single-molecule level with slow relaxation of magnetization within bi-stable doublet, a phenomenon also known as single molecule magnetism.^17^–^21^ Indeed, during the last five years several lanthanide, and especially Dy EMFs have been found to behave as single molecule magnets (SMMs).^6^,^16^,^22^–^29^

DySc_2_N@C_80_ with an icosahedral carbon cage was the first EMF proven to be a SMM.^28^ Both SQUID magnetometry and X-ray magnetic circular dichroism indicated that at low temperature the molecule exhibits magnetic hysteresis with an abrupt drop of the magnetization in zero magnetic field ascribed to the quantum tunneling of magnetization (QTM). Computational studies predicted large ligand field splitting in the range of 1500 cm^–1^ and negligible mixing of the *J*_*z*_ = ±15/2 states in the ground doublet.^9^,^10^ Higher-temperature studies with AC magnetometry were not yet possible at that time, so the barrier to relaxation *via* the Orbach mechanism involving excited LF states could not be determined. Yet in another Dy-Sc EMFs, Dy_2_ScN@C_80_, a large barrier of 1735 K due to the Orbach relaxation *via* the 5th Kramers doublet was obtained by us recently in good agreement with CASSCF calculations.^29^ The magnetization behavior of the dinuclear Dy_2_ScN@C_80_ SMM is different from that of the single-ion magnet (SIM) DySc_2_N@C_80_ in that the former does not show zero-field QTM. Exchange and dipolar interactions between the non-collinear magnetic moments of the two Dy ions in Dy_2_ScN@C_80_ create an additional barrier, which prevents QTM in zero field.^16^ Several other dinuclear Dy-EMF SMMs have been studied, including the carbide clusterfullerenes Dy_2_TiC@C_80_, Dy_2_TiC_2_@C_80_, and Dy_2_C_2_@C_82_,^6^,^26^ the sulfide clusterfullerenes Dy_2_S@C_72_ and Dy_2_S@C_82_,^6^ and the dimetallofullerene Dy_2_@C_80_(CH_2_Ph) with a single-electron Dy–Dy bond, featuring giant exchange interactions and a high blocking temperature of the magnetization.^23^ Field-induced single molecule magnetism was also found in non-Dy EMFs, like HoSc_2_N@C_80_^27^ and TbCN@C_2*n*_ (2*n* = 76, 82).^22^,^24^ The relaxation of magnetization in non-Kramers Tb and Ho SIMs is too fast to produce hysteresis, at least at temperatures above 1.8 K. With AC magnetometry, however, an evidence of millisecond-long relaxation times in finite magnetic fields at temperatures below 5–6 K could be observed.

The highest blocking temperature of magnetization at 60 K and the highest anisotropy barrier of ∼1800 K among all SMMs so far was observed in a dysprosocenium-based Dy-SIM.^30^,^31^ This Dy-SIM emphasizes the importance of ligand field design as well the diminishment of under-barrier relaxation pathways. As similarly large LF splitting is predicted for DySc_2_N@C_80_^9^,^10^ and indeed observed in Dy_2_ScN@C_80_,^29^ a detailed analysis of the relaxation of magnetization in the former is due. Whereas dinuclear EMF-SMMs have already been studied reasonably well, the basic understanding of the EMF-SIMs, including DySc_2_N@C_80_, is still lacking for high temperature regime, as well as for zero-field tunneling.

Since the discovery of SMM behavior in the Mn_12_ complex in 1993,^32^ QTM has been recognized as one of the most characteristic and fascinating features of SMMs.^33^ Magnetic moments of transition metals in such cluster-SMMs are coupled into giant-spin states of moderate total magnetic anisotropy, giving multiple QTM transitions at different magnetic fields due to avoided level crossing. Extended studies of these processes laid the foundation for further understanding of SMMs.^34^–^37^ With the advent of lanthanide-SMMs,^20^,^21^,^38^–^40^ a diminished role of QTM might be expected. Lanthanide ions usually feature much stronger magnetic anisotropy, hence preventing avoided level crossing in the normally accessible range of magnetic fields (note that avoided level crossing is still possible for multinuclear systems,^41^ or when coupling to nuclear spin can be resolved, as in Ho,^40^,^42^,^43^ Tb,^44^ or isotopically enriched ^163^Dy compounds;^45^ in such system, several QTM steps can be observed in magnetization curves at low temperatures). According to Kramers theorem, the spin states of the Dy^3+^ ion are two-fold degenerated and are time reversed of each other. Time-inversion symmetry “protects” the components of the doublet and forbids QTM in zero-field. Yet, zero-field QTM is very ubiquitous in lanthanide SIMs and can easily be observed in magnetization curves as a drop of magnetization in zero magnetic field, leading to a characteristic “butterfly” shape of hysteresis curves.^46^–^48^ It is believed that the tunneling gap in Dy can be induced by local dipolar and hyperfine fields acting on the transverse components of the *g*-tensor. Application of a finite magnetic field is usually sufficient to quench the QTM, yet it is rather surprising that QTM is so efficient in Dy-SIMs.

Electron-nuclear spin–spin interactions might appear as a natural explanation for QTM in Kramers-ion lanthanide SIMs. For instance, Dy has four similarly abundant main isotopes with nuclear spins of +5/2 (^161^Dy, 18.9%), 0 (^162^Dy, 25.5%), –5/2 (^163^Dy, 24.9%), and 0 (^164^Dy, 28.3%). Indeed, distinct influence of isotopic composition on the QTM was reported for several Dy-SMMs,^45^,^49^,^50^ yet the influence of intermolecular interactions in QTM-driven relaxation of magnetization is much more pronounced.

Tunneling of magnetization is possible when the Zeeman splitting of the two related spin states is smaller than the tunneling gap. However, dipolar interactions between spins create a distribution of the local magnetic fields acting on individual molecules. As a result, the level alignment required for tunneling is met only for a tiny fraction of molecules, thus formally precluding the tunneling. Yet, the experimental facts are very clear – QTM is a very efficient process in many SMMs. Prokof’ev and Stamp developed a theory showing that when spins flip, dipolar fields are readjusting bringing more spins into resonance.^51^ This theory motivated a handful of studies of the dipolar field distribution and its influence on the QTM relaxation rate for transition metal SMMs.^34^ The “Hole-digging” technique developed by Wernsdorfer *et al.* gave especially illustrative results.^52^,^53^ Simulations by Garanin *et al.* showed that adjusting dipolar fields may create a tunneling front, which can propagate through a sample.^54^,^55^ This process was dubbed as dipolar avalanche or cold deflagration in analogy to thermal avalanches well described for transition metal SMMs.^56^,^57^ Long *et al.* suggested that fast zero-field QTM in Er(COT)_2_ proceeds *via* dipolar avalanches,^58^ and if so, it is natural to propose similar effects in other lanthanide SIMs. Nevertheless, detailed studies of the influence of local dipolar field distribution for lanthanide SMMs are still missing despite the strong interest in lanthanide SMMs during the last decade and despite the general understanding of the important role that cooperative effects play in their magnetic properties.^49^,^58^–^64^

The importance of intermolecular interactions in QTM raises the question of to which extent the properties of SIMs measured in bulk samples are indeed single molecule properties. The influence of this factor can be partially diminished by diluting the sample in a diamagnetic matrix. For instance, the use of Y analogs to lanthanide compounds is a natural choice and is hence rather popular.^49^,^50^,^58^,^60^,^61^,^65^–^67^ In the first study of single molecule magnetism in DySc_2_N@C_80_, Westerström *et al.* showed that dilution of the sample with C_60_ considerably increased relaxation times.^28^ Wang *et al.* recently found that adsorption of DySc_2_N@C_80_ inside the metal–organic framework MOF-177 reduced the QTM, and the authors hypothesized that spin–phonon interactions in the MOF lattice might be the reason.^68^ Yet, detailed analysis of the relaxation of magnetization in this archetypical fullerene SMM is still missing. Here we present an extended study of magnetic relaxation in DySc_2_N@C_80_ at different temperatures, in varied magnetic fields, and in different molecular arrangements. We study relaxation of magnetization in powder and single-crystalline samples of DySc_2_N@C_80_ with a particular focus on the influence of intermolecular interactions and the effect of dilution on these properties. The manuscript is organized as following: at first, molecular and crystal structure of DySc_2_N@C_80_ is clarified by single-crystal X-ray diffraction. Then magnetization behavior and relaxation are studied for non-diluted powder samples, and for fullerenes diluted in three different non-magnetic matrices. Field dependence and zero-field relaxation are then studied for single-crystals of DySc_2_N@C_80_ co-crystallized with Ni(ii) octaethylporphyrin (Ni^II^(OEP) hereafter). AC magnetometry is used then to get access to relaxation of magnetization in a broader temperature range. Finally, a theoretical framework for studying spin dynamics with dissipation is put forward.

## Synthesis and single-crystal X-ray diffraction

DySc_2_N@C_80_-I_h_ (I_h_ denotes the icosahedral isomer of the C_80_ cage; the symbol will be omitted as only EMFs with C_80_-I_h_ cage will be discussed hereafter) was obtained by arc-discharge synthesis using the procedure described in earlier.^29^ In brief, graphite rods packed with a mixture of Dy_2_O_3_, Sc_2_O_3_, and guanidine thiocyanate are subjected to evaporation in He atmosphere (180 mbar). CS_2_ extraction of the soot obtained by arc discharge yields a mixture of fullerenes, with Sc_3_N@C_80_, DySc_2_N@C_80_, and Dy_2_ScN@C_80_ as the main components. The mixture was further separated by multi-step HPLC to give pure DySc_2_N@C_80_ (see ESI,† for details of separation steps and mass-spectrometric characterization). The fullerene is soluble in many organic solvents (in particular, toluene and CS_2_ solutions were used to prepare drop-casted powder samples for magnetometry).

Single crystals of DySc_2_N@C_80_ were grown by co-crystallization with Ni^II^(OEP). Two batches of crystals were prepared by layering a benzene solution of Ni^II^(OEP) over a solution of DySc_2_N@C_80_ in either CS_2_ or toluene. Black block crystals with a size of up to 500 μm formed after the fullerene and Ni^II^(OEP) solutions diffused together over a period of *ca.* one month. X-ray diffraction data collection for the crystals was carried out at 100 K at the BESSY storage ring (BL14.3, Berlin-Adlershof, Germany)^69^ using a MAR225 CCD detector, *λ* = 0.89429 Å. Processing of the diffraction data was done with XDSAPP2.0 suite.^70^ The structure was solved by direct methods and refined using all data (based on *F*^2^) by SHELX 2016.^71^ Hydrogen atoms were located in a difference map, added geometrically, and refined with a riding model. The crystal data and collection parameters are presented in Table S1 (ESI†). CCDC ; 1587626 and ; 1587784.†

The crystals grown in CS_2_/benzene solvents have the structural formula DySc_2_N@C_80_·Ni^II^(OEP)·2(C_6_H_6_) (type **I** hereafter), whereas in the toluene/benzene system both solvents are found in the lattice giving the formulae DySc_2_N@C_80_·Ni^II^(OEP)·0.72(C_6_H_6_)·1.28(C_7_H_8_) (type **II** hereafter). In both crystals, the carbon cage is fully ordered and is coordinated to the Ni^II^(OEP) molecule. The nearest cage carbon–Ni distances are 2.84(1) Å in **I** (Ni1P···C1A) and 2.87(1) Å in **II** (Ni1P···C19A).

In the crystal **I**, the DySc_2_N cluster is well ordered, only Dy has two sites with fractional occupancies of 0.948 and 0.052. In the crystal **II** the DySc_2_N cluster is disordered between two positions. The main one (fractional occupancy of 0.817) is identical to that in the crystal **I**, whereas in the minor one, positions of Dy and one of the Sc atoms are flipped. Thus, in both crystals the main DySc_2_N site has two Sc ions directed towards the Ni^II^(OEP) molecule, and the Dy ion facing the opposite side of the fullerene cage. A similar arrangement of the lanthanide–scandium mixed MSc_2_N cluster was reported in other co-crystals of MSc_2_N@C_80_ (M = La, Ce, Gd, Tb, and Er) with Ni^II^(OEP) or Co^II^(OEP).^72^–^75^

**Fig. 1:**
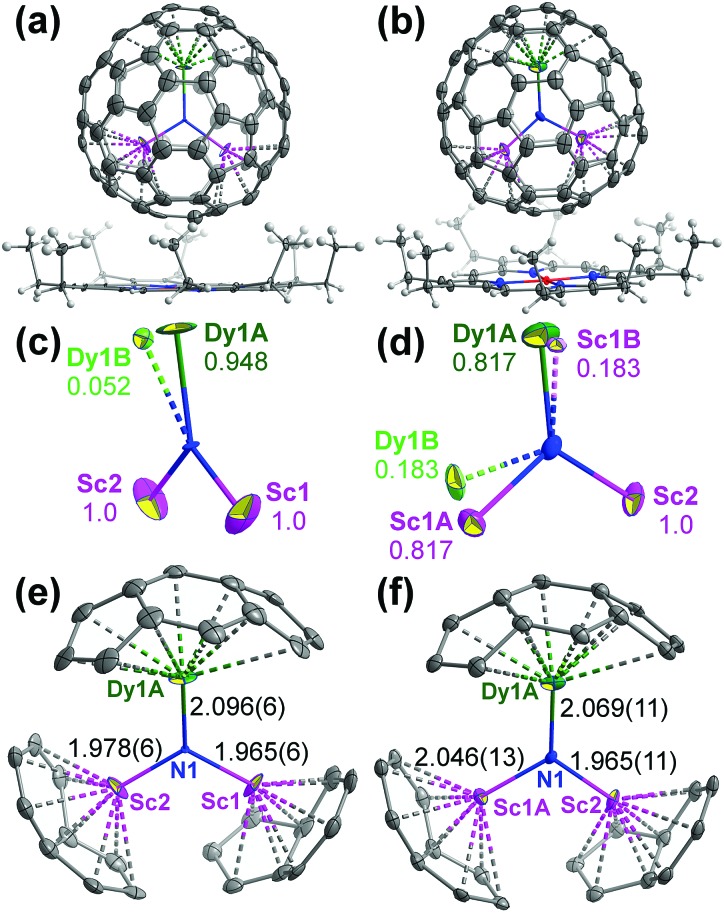
(a and b) Mutual orientations of the DySc_2_N@C_80_ and Ni^II^(OEP) molecules in crystals **I** (a) and **II** (b), only the main site of the endohedral cluster is shown, the displacement parameters are shown at the 30% (a) and 50% (b) probability levels; (c and d) major and minor metal sites in the DySc_2_N cluster in crystals **I** (c) and **II** (d); (e and f) the main DySc_2_N sites in **I** (e) and **II** (f), coordinated cage carbons (metal–carbon distances less than 2.98 and 2.84 Å for Dy and Sc, respectively), and metal–nitrogen bond lengths (in Å). Bond angles in the DySc_2_N cluster are 113.2(3)° Sc1–N1–Sc2, 124.0(3)° Sc1–N1–Dy1A, 122.7(3)° Sc2–N1–Dy1A in **I**, and 108.9(6)° Sc1A–N1–Sc2, 123.7(5)° Sc1A–N1–Dy1A, 127.4(6)° Sc2–N1–Dy1A in **II**.

Fig. 1 shows the DySc_2_N cluster in both crystals along with metal-coordinated carbon atoms of the fullerene cage and selected geometry parameters. The Sc–N–Sc angle is 113.2(3)°, and the Sc–N–Dy angles are 122.7(3) and 124.0(3)°. The sum of angles at nitrogen is 359.9°, and the DySc_2_N cluster is essentially planar. The Dy–N bond in the main cluster site in **I** is 2.096(6) Å long, whereas the two Sc–N bonds are 1.965(6) and 1.978(6) Å (the overlap of two DySc_2_N sites in **II** makes the bond length parameters for this structure less reliable). In Dy_2_ScN@C_80_, the Dy–N bond is a bit shorter, 2.078(6) Å,^29^ whereas in Dy_3_N@C_80_, the Dy–N bond distances are found in the range from 2.004(8) to 2.068(8) Å^76^ or from 2.017 to 2.087 Å.^77^ The Dy–N bonds in EMFs are strikingly shorter than the typical Dy–N bonds in Dy coordination compounds (Table S2, ESI†). Except for a few examples with relatively short Dy–N bonds (the shortest non-fullerene Dy–N bond length is 2.14 Å),^78^–^82^ the majority of such compounds feature Dy–N bond lengths of 2.4–2.6 Å.^83^

**Fig. 2:**
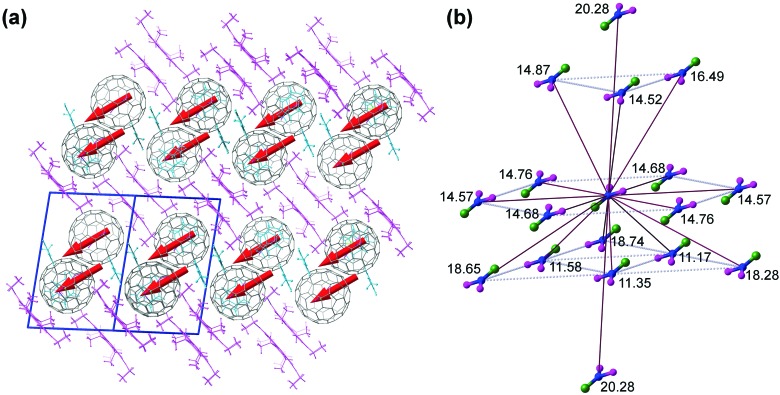
(a) Packing of molecules in the DySc_2_N@C_80_·Ni^II^(OEP)·0.72(C_6_H_6_)·1.28(C_7_H_8_) crystal (**II**). Fullerenes are shown in grey, porphyrin molecules in magenta, and solvent molecules in cyan. Red arrows indicate positions of Dy atom and directions of the Dy–N bonds. Blue lines denote the unit cell. (b) The closest fullerene neighbors (inter-fullerene distance 20 Å or less) in the crystal **II** (only major sites of DySc_2_N clusters are shown: Dy is green, Sc is magenta, N is blue, the numbers are N···N distances to the “central” molecule in Å).

Although crystals **I** and **II** have different space groups (*C*2/*c* and *P*1, respectively), the packing of fullerene molecules in the crystals is very similar. Since our magnetic measurements discussed below were performed for the crystal **II**, its crystal structure is discussed next. Fig. 2 shows packing in the crystal **II** and the closest inter-fullerene distances. Fullerenes are arranged in the slightly distorted hexagonal close packed layers, which form A–B stacked bilayers. The space between bilayers is filled with Ni^II^(OEP) molecules, whereas solvent molecules occupy voids in A–B stacks. In the crystal **II**, the closest distances between fullerenes, measured as a distance between N atoms of DySc_2_N cluster, within each hexagonal layer are 14.57, 14.68, and 14.76 Å. The distance between A and B layers in the bilayer is only 7.58 Å, so that the closest distances between fullerene molecules from layers A and B are 11.17, 11.35, and 11.58 Å. The distance between layers A and B from different bilayers is 12.55 Å, and the closest inter-bilayer distances between fullerene molecules are 14.52, 14.86, and 16.49 Å. The DySc_2_N cluster is tilted with respect to the hexagonal layers, but all Dy–N bonds are parallel (as far as the mainly occupied site is concerned, Fig. 2). Since the magnetic moment of DySc_2_N@C_80_ is oriented along the Dy–N bond, parallel alignment of Dy–N bonds implies that the crystal has a macroscopic easy axis of magnetization (Fig. 2a). The presence of the less abundant sites should result in a slight misalignment of the effective easy axis of magnetization of the crystal from the main Dy–N direction.

For the magnetic dilution studies discussed below, co-crystallization with Ni^II^(OEP) was also performed for a mixture of DySc_2_N@C_80_ (10%) and non-magnetic Lu_3_N@C_80_ (90%) using CS_2_ as a solvent for the fullerenes. The refinement of Sc and Dy positions cannot be performed in the presence of 90% of Lu, however positions of the Lu atoms as well as the fullerene cage and the Ni^II^(OEP) molecule can be determined (Fig. S4, ESI†). Site occupancies for Lu of *ca.* 0.9 correspond to the composition of the fullerene mixture. The fullerene cage of the Lu_3_N@C_80_ molecule is ordered by Ni^II^(OEP). The Lu_3_N cluster can be refined to three sites very close to one another with comparable occupancies of 0.316, 0.283, and 0.268. The distances between fractionally occupied sites are 0.5–0.7 Å, and in principle it is possible to refine the three sites as one but with strongly elongated ellipsoid for each Lu atom. The orientation of the Lu_3_N cluster with respect to the Ni^II^(OEP) molecule is the same as in many other M_3_N@C_80_·Ni^II^(OEP) crystals.^77^ The presence of DySc_2_N@C_80_ molecules in these crystals is evident from magnetic measurements discussed below. The packing mode of fullerene molecules is very similar to that in the crystal **II**. Taking into account the strong orienting effect imposed on the DySc_2_N cluster by the Ni^II^(OEP) molecule in crystals **I** and **II**, a similar alignment of the cluster and hence the presence of a macroscopic anisotropy axis can be expected in the diluted crystal as well.

### Magnetization behavior of powder samples

**Fig. 3:**
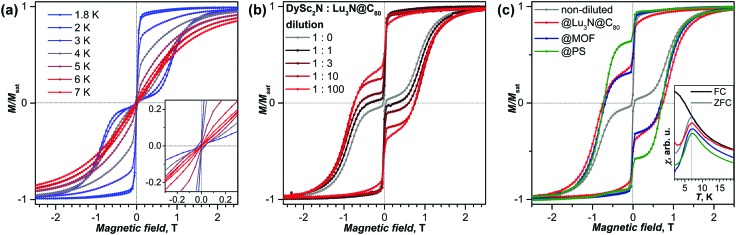
(a) Magnetization curves of DySc_2_N@C_80_ powder samples measured at different temperatures between 1.8 and 7 K, the inset shows the curves for *T* = 3–7 K near zero field. (b) Magnetization curves of DySc_2_N@C_80_ diluted with Lu_3_N@C_80_ in different ratios, *T* = 1.8 K. (c) Magnetization curves of DySc_2_N@C_80_ diluted in different diamagnetic matrices (Lu_3_N@C_80_, MOF DUT-51(Zr), and polystyrene (PS)), *T* = 1.8 K; the inset shows the peak in the temperature dependence of the magnetic susceptibility *χ* used of the determination of the blocking temperature *T*_B_ (the black curve was measured during cooling the non-diluted sample in a field of 0.2 T (FC), the other curves were measured during increase of the temperature for the samples cooled in zero field (ZFC); the susceptibility curves for different dilution matrices are shown with a slight offset, temperature sweep rate is 5 K min^–1^). All magnetization curves were measured with a sweep rate of 2.9 mT s^–1^.

Fig. 3a shows magnetization curves of a microcrystalline powder sample of DySc_2_N@C_80_ obtained by drop-casting from a CS_2_ solution. With a sweep rate of 2.9 mT s^–1^, the sample shows hysteresis up to 7 K. The blocking temperature of magnetization *T*_B_ = 7 K is determined as the temperature of the peak of the magnetic susceptibility measured for the zero-field cooled sample with a temperature sweep rate of 5 K min^–1^.

The sudden drop of the magnetization on crossing zero magnetic field results from the fast QTM process. This process prevents the study of the intrinsic relaxation time of DySc_2_N@C_80_ in zero field, and to reduce its influence we attempted to increase the distance between the magnetic molecules by dilution. Three matrices were applied in this work to achieve this goal: dilution with diamagnetic Lu_3_N@C_80_, adsorption into voids of the porous Metal Organic Framework (MOF) DUT-51(Zr),^84^ and dispersion in the polymer polystyrene.

The mixing with diamagnetic fullerenes is the most straightforward and well controlled dilution approach, albeit requiring large amounts of diamagnetic fullerenes to achieve a strong dilution effect. Westerström *et al.* showed that mixing of DySc_2_N@C_80_ with C_60_ substantially increased relaxation times,^28^ but a more detailed analysis was not possible at that time. The use of a fullerene with a different carbon cage for dilution increases the probability of a separate crystallization of two fullerenes. To avoid this, in this work we decided to use Lu_3_N@C_80_ with the same fullerene cage as in DySc_2_N@C_80_. Fig. 3b shows the hysteresis curves measured at 1.8 K for a series of mixed-fullerene samples in dependence on the ratio between DySc_2_N@C_80_ and Lu_3_N@C_80_ from 1 : 1 to 1 : 100. The samples were obtained by mixing solutions of two fullerenes and then drop-casting. The progressive influence of dilution on the QTM step can be immediately recognized: whereas the non-diluted sample shows a zero-field drop of magnetization to almost zero, dilution opens the gap in hysteresis, and the size of the gap is increasing with the degree of dilution. However, even for the 1 : 100 dilution, still *ca.* 60% of the DySc_2_N@C_80_ molecules lose their magnetization *via* QTM. For comparison, fast zero-field QTM in [Er(COT)_2_]^–^ diluted in 1 : 85 ratio with Y analog was observed for *ca.* 30% of the molecules.^58^ Further increase of dilution by Lu_3_N@C_80_ would require much larger amounts of the fullerene, and appears not very practical.

MOFs can serve as an inert diamagnetic matrix and therefore also affect QTM.^68^,^85^–^87^ In this work we used DUT-51(Zr), (DUT states for Dresden University of Technology), synthesized along with previously reported procedure.^84^ The diamagnetic framework is constructed of Zr_6_O_8_ clusters, interconnected by eight bent ligands dithieno[3,2*-b*;2′,3′*-d*]thiophene-2,6-dicarboxylate, resulting in 8-connected framework with **reo** topology. The framework possesses a hierarchical pores structure involving cages of 15.6, 18.8 and 24.5 Å in size and pore windows of 9 Å, which are large enough to host fullerene molecules. The powder of the desolvated DUT-51(Zr) was immersed into a toluene solution of DySc_2_N@C_80_, and the decrease of the fullerene concentration over time was followed with the help of Vis-NIR spectroscopy (see Fig. S5 and S6, ESI† for details). After a fast drop of the fullerene concentration in solution caused by adsorption into the voids of DUT-51(Zr) during the first 24 hours, a steady concentration in the solution was achieved after *ca.* 100 hours. The fullerene solution over the DUT-51(Zr) was refreshed four times reaching, in the end, the fullerene/MOF mass ratio of *ca.* 1 : 100 (0.18 mg of DySc_2_N@C_80_ was adsorbed by 19.6 mg of DUT-51(Zr)). The magnetization curve of the DySc_2_N@C_80_@DUT-51 sample shown in Fig. 3c has similar features to the sample diluted with the diamagnetic fullerene. We conclude therefore that the influence of MOF encapsulation on QTM in DySc_2_N@C_80_ such as observed by Wang *et al.*^68^ is most probably a dilution effect.

The most pronounced effect of dilution on QTM in DySc_2_N@C_80_ is observed for the fullerene dispersed in a film of polystyrene. Solutions of DySc_2_N@C_80_ and polystyrene in toluene or CS_2_ were mixed, and then drop-casted onto a glass slide giving a solid film after evaporation of the solvent. The most pronounced suppression of the QTM (Fig. 3c) was achieved by using *ca.* 1 : 10 000 fullerene : polymer mass ratio and CS_2_ as a solvent. The use of toluene as a solvent resulted in a partial phase separation and formation of fullerene microcrystals visible by optical microscopy (Fig. S7, ESI;† see also ref. 88 for the detailed study of fullerene crystallization during the preparation of fullerene/polymer films). Such samples also showed partial suppression of QTM (Fig. S7, ESI†) but to a much lesser extent than found for the sample shown in Fig. 3c. The QTM-induced drop of magnetization at zero field decreased to *ca.* 40%.

To summarize, all three dilution methods (diamagnetic fullerene, MOF, and dispersion in polymer) resulted in a substantial decrease of the QTM-induced zero-field drop of magnetization in the hysteresis curves of DySc_2_N@C_80_. The most pronounced influence was achieved by the use of a polymer, which appears to be a simple and efficient way of studying magnetically diluted samples. Despite the strong influence of dilution on the QTM step, the *T*_B_ values remain non-affected and remain near 7 K for all samples (Fig. 3c). Note that these values correspond to a specific magnetic field (0.2 T), in which *χ*–*T* curves were measured. Since magnetic field has a strong influence on relaxation time as discussed in the next section, different *T*_B_ values may be obtained in a different field.

### Magnetization and QTM behavior of single crystals

The relaxation time of magnetization (*τ*_m_) has a complex dependence on the magnetic field due to the possible combination of at least two field-dependent relaxation mechanisms, QTM and direct:1
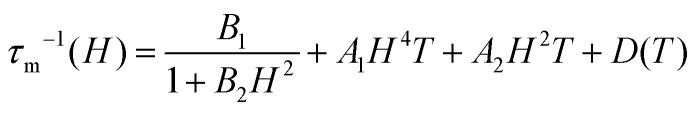
Here *H* is the field, the first term describes field-dependence of the QTM, *B*_1_ and *B*_2_ are fitting parameters, *B*_1_ is the zero-field QTM relaxation rate.^89^,^90^ The second and third terms describe the relaxation of a Kramers ion *via* the direct mechanism in the absence (∼*H*^4^) and in the presence (∼*H*^2^) of hyperfine interactions, *A*_1_ and *A*_2_ are fitting parameters. Finally, the function *D*(*T*) includes the rates of field-independent relaxation processes, such as proceeding under Orbach and Raman mechanisms.^91^

**Fig. 4:**
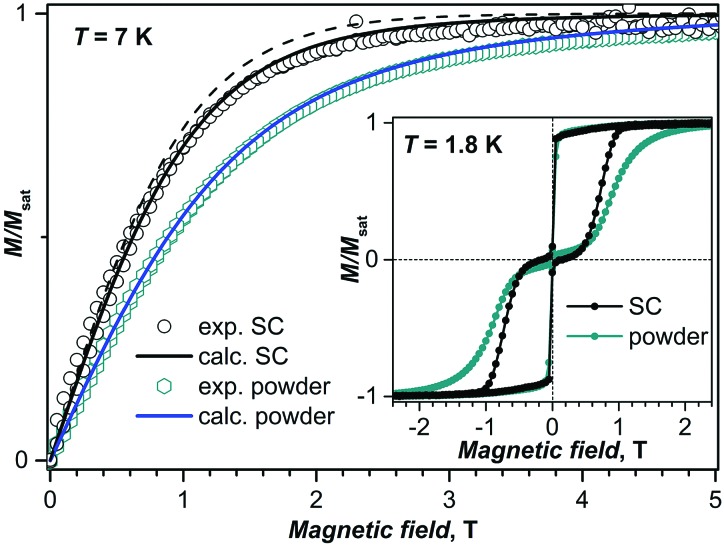
Experimental (dots) and calculated (lines) magnetization curves of the powder and aligned single-crystal **II** (SC) of DySc_2_N@C_80_ at *T* = 7 K. Calculations for the single crystal took into account the presence of two crystallographic DySc_2_N sites in the crystal **II** (solid line); the dashed line is the calculated magnetization curve for the main site only. The inset shows magnetic hysteresis curves measured at 1.8 K for the powder and for the single crystal.

Due to large single-ion anisotropy, the magnetic moment of the DySc_2_N@C_80_ molecule is aligned along its Dy–N bond. In powder samples with all possible orientations of the molecules, each molecule experiences an effective bias field, equal to the projection of the external magnetic field onto the direction of the anisotropy axis, *i.e.* external field is scaled with a cosine of the angle between the Dy–N axis and the external field direction. The field dependence of the relaxation rate in eqn (1) is then distorted by a continuous distribution of angles in a powder sample. Therefore, magnetization relaxation times were measured for the single-crystal DySc_2_N@C_80_·Ni^II^(OEP) (**II**). The rectangular block crystal was immersed into a vacuum grease on the holder with the long crystal side oriented along the field. Slow cooling of the crystal in a field of 7 Tesla resulted in its rotation and the orientation of the magnetic anisotropy axis of the crystal along the field direction (see Fig. S8 (ESI†) for more details on the alignment). Simulations of the magnetization curves measured for powder and single-crystal samples at 7 K (near the blocking temperature) using PHI code^92^ and *ab initio* ligand-field parameters for Dy in DySc_2_N@C_80_ computed at the CASSCF/RASSI level (see ESI†) showed a good agreement between experiment and theory (Fig. 4).

**Fig. 5:**
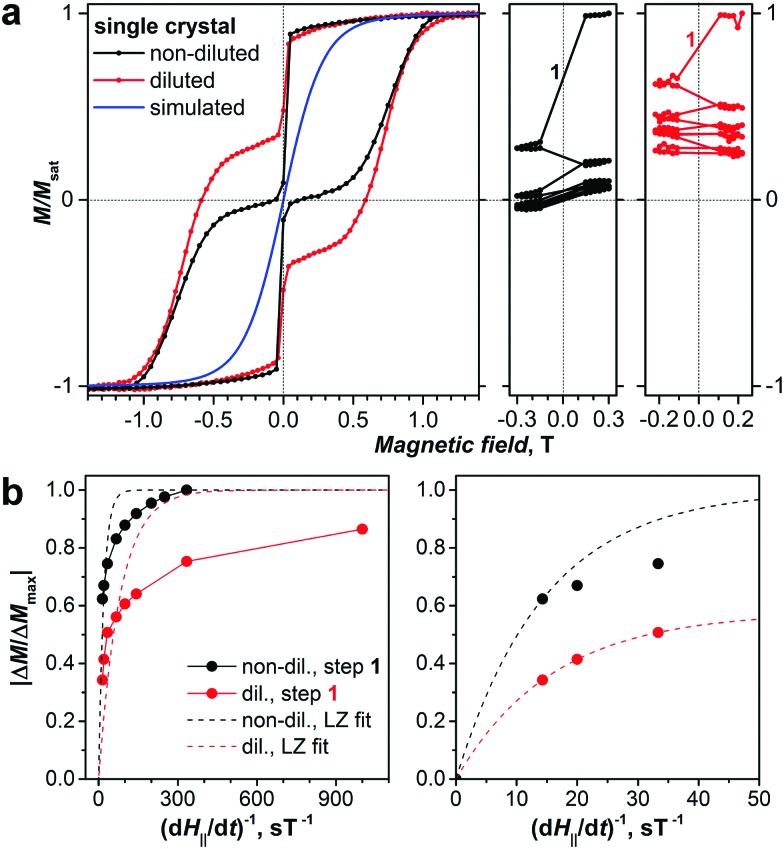
(a) Magnetization curves of diluted (red) and non-diluted (black) single crystal DySc_2_N@C_80_·Ni^II^(OEP) (**II**) at 1.8 K as compared to the simulated thermodynamic magnetization curve (blue). For the sake of better comparison, the magnetic field of the non-diluted crystal was scaled by cos(43°) to take into account misalignment of the anisotropy axis with respect to the direction of the external field. The right panels show changes of the normalized magnetization upon multiple scans in the range of [–0.3 T, +0.3 T]. (b) Sweep rate dependence of the relative magnetization drop upon crossing zero magnetic field in non-diluted (black dots) and diluted (red dots) crystals. Dashed lines represent the calculated *P*_QTM_ dependences calculated using eqn (3) for the whole set of points (left) and for the data point obtained with the fastest sweep rate (right).

In-field cooling of the diluted DySc_2_N@C_80_/Lu_3_N@C_80_·Ni^II^(OEP) crystal (1 : 9 dilution) did not cause its reorientation, presumably because the torque is not sufficiently strong for the diluted crystal. However, comparison of the magnetization curves with those of the oriented single crystal DySc_2_N@C_80_·Ni^II^(OEP) and to the simulated curves showed that the diluted crystal also has a preferred orientation of the DySc_2_N cluster. This analysis also showed that the anisotropy axis of the diluted crystal in these measurements was misaligned from the external field direction by *α* = 43°. Scaling the field axis by cos(*α*) gives almost a perfect coincidence of the normalized magnetization curves for the diluted and non-diluted crystals (except for the zero-field range, see Fig. S9 (ESI†) for more details). Therefore, the external magnetic field values are scaled by cos(43°) = 0.73. Similar to what was observed in the measurements on powder samples, the single crystal of DySc_2_N@C_80_ diluted with Lu_3_N@C_80_ showed a considerable decrease of the QTM-induced drop of magnetization in zero magnetic field (Fig. 5).

### Landau–Zener theory

QTM in SMMs is often analyzed using the Landau–Zener theory. According to this model, the probability of the system to tunnel when crossing the resonance (zero field in our case) is determined by the formula:^93^,^94^
2
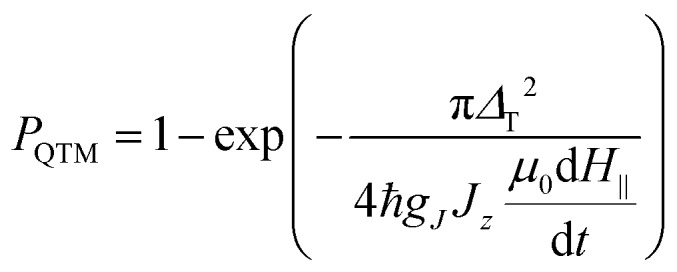
where *Δ*_T_ is the tunneling gap, *g*_*J*_*J*_*z*_ is 10 *μ*_B_ for Dy^3+^ in the ground state, and *μ*_0_d*H*_II_/d*t* is the magnetic sweep rate.

To measure *P*_QTM_ with our SQUID-VSM magnetometer, we first magnetized the crystal to saturation, then reduced the field down to +0.3 T, and then ramped it back and forth in the [–0.3 T, +0.3 T] range (similar experiments were performed in the studies of fast QTM in transition metal clusters^34^–^36^). In these scans, magnetization at each point was measured for 5–10 s followed by a step of 30 mT to the next point, except for the range between +0.15 and –0.15 T, which was swept in a single step. The rate of the jump around zero-field was varied from 70 mT s^–1^ (the highest sweep rate of the magnetometer) to 1 mT s^–1^. Fig. 5 shows the variation of the magnetization of the non-diluted and diluted DySc_2_N@C_80_ crystals during these ramps measured with the 70 mT s^–1^ zero-field crossing. For the non-diluted crystal, a stepwise drop at each sweep is observed, quickly leading to a complete loss of magnetization after three cycles. A rapid loss of the magnetization on crossing zero field is also observed in the diluted crystal. However, upon reaching *ca.* 40% of the saturation value after a few cycles, the magnetization stabilizes and shows much slower decay at further scans.

The probability *P*_QTM_ is then defined as the ratio |Δ*M*/Δ*M*_max_|, where Δ*M* is the drop of the magnetization between the points of +0.15 and –0.15 T, and Δ*M*_max_ is the drop of the magnetization measured with the slowest sweep rate. For the non-diluted crystal Δ*M*_max_ is slightly larger than the magnetization at saturation *M*_sat_. For the diluted crystal the sweep rate dependence does not reach the limit, hence we simply used Δ*M*_max_ ≈ *M*_sat_. Fig. 5 shows the sweep rate dependence of *P*_QTM_. For the non-diluted sample the values are above 0.6 even for the fastest sweeps. For the diluted crystal the values are much smaller, but their variation with the sweep rate confirms that the sample behaves inhomogeneously, *i.e.* that the QTM is slowing down dramatically after some part of the sample tunnels.

Our measurements showed that neither for non-diluted nor for diluted DySc_2_N@C_80_ crystals the sweep rate dependence of the *P*_QTM_ follows the Landau–Zener formula (see Fig. 5b for the fits). One of the prerequisites of the Landau–Zener theory to be applicable is that the tunneling gap is not changing during the measurement. Presumably, the change of the magnetization state in a large part of the sample strongly affects the local distribution of dipolar fields (see below) and hence substantially changes the tunneling gap. Therefore, only a conservative estimation of the *Δ*_T_ value can be done using the smallest *P*_QTM_ values. For the non-diluted sample, it can be seen that eqn (2) using the *Δ*_T_ value estimated for the fastest sweep-rate point shows strong deviations from the points measured with slower sweep rates. For the diluted crystal, we took into account the non-uniform behavior and multiplied eqn (2) with a scaling factor. With the factor of 0.58, *i.e.* if we consider that only 58% of the sample is described by this equation, the first three *P*_QTM_ points appear to follow eqn (2), but at slower sweep rates the deviation is increasing. The approximate values of *Δ*_T_ estimated from these tentative fits are very large, 0.01–0.02 cm^–1^, which is several orders of magnitude larger than usually observed in transition metal SMMs. For comparison, this gap corresponds to Zeeman splitting in the ground state Kramers doublet of Dy^3+^ induced by a magnetic field of 1 mT.

### Magnetic field dependence of relaxation times

To measure relaxation times of the magnetization (*τ*_m_) with DC magnetometry, the samples are first magnetized in the field of 3 Tesla (which is beyond the point where hysteresis is closed), then the field is ramped as fast as possible to the desired value, and then the decay of magnetization is recorded over time. Reliable determination of extremely long relaxation times (over 10^5^ s) found in finite fields of 0.05 < *μ*_0_*H* < 0.4 T requires over 10 hours of decay measurement. The curves are then fitted with a stretched exponential function. This procedure provides a trustworthy estimations of relaxation times longer than 100 s. The time lag in the beginning of the measurements due to the field stabilization precludes an accurate measurement of faster relaxation processes. ESI† gives a detailed description of the technical aspects of the measurements of relaxation times and determined values.

According to eqn (1), one can expect two regimes in the magnetic field dependence of *τ*_m_. QTM is prevalent in small finite fields. With the increase of the field, *τ*_m_ grows while QTM should be gradually diminished by the growing energy difference between the states of the opposite spin. Once QTM is completely switched off, the direct mechanism is expected to become the dominant one, at least at low temperatures, and thus relaxation times shorten with further increase of the magnetic field.

**Fig. 6:**
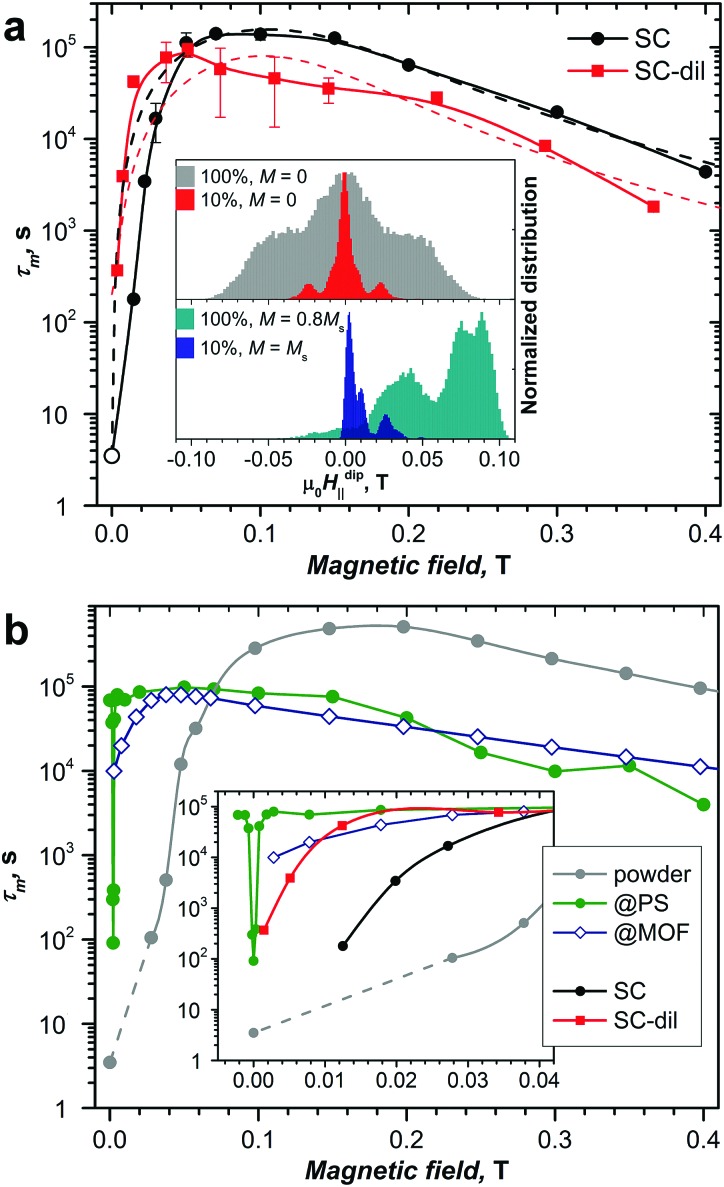
(a) Relaxation times of magnetization measured at 1.8 K for diluted (red) and non-diluted (black) single crystals (SCs) as a function of magnetic field. Solid lines are spline-interpolated and are only shown to guide the eye, dashed lines are fits with eqn (1). The inset shows the simulated distribution of *H*dip‖ in non-diluted (100%) and diluted (10%) crystals at different magnetization states (*M*_s_ is the magnetization of the fully polarized sample); (b) relaxation times of magnetization measured at 1.8 K for non-diluted powder (gray) as well as for diluted samples in MOF (blue) and polystyrene (PS, green). The inset zooms into the small field range.

Fig. 6 shows the field dependence of *τ*_m_ obtained for non-diluted and diluted crystals at the temperature of 1.8 K. For the non-diluted crystal, relaxation in the field smaller than 13 mT is too fast to be measured reliably. The increase of the field beyond this value leads to a fast increase of *τ*_m_ over several orders of magnitude, with the maximum value of 1.4 × 10^5^ s in fields of 70–100 mT. In the diluted crystal, the increase of the relaxation time with magnetic field is even sharper, and the maximum value of *τ*_m_ = 0.9 × 10^5^ s is reached already in the field of 50 mT. In stronger magnetic fields, *τ*_m_ values decrease gradually as expected for the relaxation *via* the direct mechanism (eqn (1)). In this relaxation regime, *τ*_m_ values of the diluted crystal are systematically shorter by a factor of 2–3.

Similar trends are obtained for the powder samples. The non-diluted powder of DySc_2_N@C_80_ shows an increase of the relaxation time from 3.5 s in zero field (estimated by AC magnetometry, see below) up to the maximum of 5 × 10^5^ s in a field of 150–200 mT. Thus, the field-induced increase of the relaxation time is slower in the powder sample than in the single crystal. Diluted powder samples feature a much sharper increase of *τ*_m_ values with the field. In DySc_2_N@C_80_@MOF, the longest *τ*_m_ value of 0.8 × 10^5^ s is found in the field of 40 mT. But the most pronounced influence of dilution on the *τ*_m_–*H* dependence is observed for DySc_2_N@C_80_ in the polystyrene matrix. In this sample, the long relaxation times of 0.7 × 10^5^ s are measured down to 2 mT; in a field of 1 mT the *τ*_m_ value drops to 0.4 × 10^5^ s, and only below that field the relaxation time decreases very fast to the value of less than 100 s in zero field. Importantly, the sharp drop of magnetization in zero field involves the whole sample, and not just a fraction of it. Such a sharp resonance can be easily missed in common commercial magnetometers due to remnant magnetic fields of up to several mT which tend to appear after the superconducting magnet is ramped fast from several Teslas down to zero in a linear mode. In fact, we could locate the resonance in our polystyrene-diluted sample only when careful measurements with small sub-mT steps were performed near zero field.

Based on these data, we can surmise the following points. At 1.8 K, the relaxation times measured near zero field and at higher fields are different by several orders of magnitude. Such a variation of the relaxation rate with the field is not unusual for SMMs with zero-field QTM because application of a finite field brings the spin levels out of resonance. However, the threshold field required to completely switch the QTM off strongly depends on the dilution state of the sample and varies from 150 mT for the non-diluted powder to less than 1 mT for the strongly-diluted DySc_2_N@C_80_ in polystyrene. Likewise, ordering also has a strong effect on the *τ*_m_–*H* dependence, and the single-crystalline DySc_2_N@C_80_ requires half the field to quench QTM compared to the powder sample.

Our attempts to fit *τ*_m_ values measured for single crystals with eqn (1) gave only poor agreement in the low-field range (Fig. 6). The strong variation of relaxation times with dilution indicate that the local fields created by dipole–dipole interactions between neighboring Dy centers play a crucial role in the relaxation of magnetization in small magnetic fields, and that the field dependence of the QTM relaxation rate most probably reflects the internal distribution of dipolar fields in the sample. Since the dipolar field scales with the distance as *R*^–3^, the contribution of the closest neighbors should be especially important. The inter-fullerene distances in the DySc_2_N@C_80_·Ni^II^(OEP) crystal can be as short as 11.2–11.6 Å (Fig. 2b). Furthermore, the two shortest Dy···Dy distances in the crystal **II** are only 9.12 and 9.24 Å. If we model a crystal by the fragment shown in Fig. 2b, define *z* axis as aligned along the Dy–N bond, and count contributions from all molecules in the fragment, then the longitudinal and transverse components of the cumulative dipolar field acting on the central Dy ion in the perfectly ordered and fully magnetized crystal are *H*dip‖ = 105 mT and *H*dip⊥ = –62 mT. When the spins start to flip (*i.e.* magnetization is decreasing), the dipolar fields in the sample become less uniform. Fig. 6a shows numerical simulations for the distribution of *H*dip‖ for the magnetization equal to 80% of the saturated value and for the fully demagnetized crystal. In the latter, the distribution is symmetric and is largely confined in the [–90 mT, +90 mT] range, whereas in the partially magnetized sample the distribution is strongly asymmetric and is *ca.* half as broad. In the diluted sample, many neighboring spins are “missing”, which strongly changes the distribution of dipolar fields. Simulations of *H*dip‖ for the 1 : 9 dilution are also shown in Fig. 6a. In the fully magnetized diluted crystal, the fraction of spins experiencing the dipolar field stronger than 50 mT is negligible, and the maximum of the distribution is close to zero field. In the fully demagnetized diluted sample the distribution becomes symmetric with respect to *H*dip‖ = 0, but the width, which is *ca.* 10 times narrower than for the non-diluted crystal, remains almost the same. Additional peaks observed near ±23 mT correspond to the nearest neighbors: Dy ions from the neighboring fullerene molecules create a longitudinal dipolar field of *H*dip‖ = 23 mT and a transverse field of *H*dip⊥ = –8 mT.

The longitudinal dipolar field distributions in diluted and non-diluted samples provide a clear explanation for the field dependence of the magnetic relaxation in the QTM-like regime near zero field. The QTM regime is completely switched off if the external bias field is exceeding the width of the dipolar field distribution. Hence non-diluted samples show the broadest QTM resonances (Fig. 6), and the increase of the width in the powder sample in comparison to the single crystal is caused by the random distribution of the anisotropy axes of different DySc_2_N@C_80_ molecules (*versus* preferential alignment along the field direction in the single crystal). Once the external field is smaller than the width of *H*dip‖, it induces QTM in a part of the sample. Furthermore, the smaller the external field, the more molecules are brought into resonance (note that with decreasing magnetization, the maximum of the dipolar field distribution is moving closer to zero). In the polystyrene-diluted DySc_2_N@C_80_, the residual dipolar fields should be very small, hence the narrow QTM resonance in Fig. 6b. In fact, when dilution is strong, the hyperfine fields cannot be ignored anymore. In addition to the Dy isotopes ^161^Dy (*I* = 5/2, 18.9%) and ^163^Dy (*I* = –5/2, 24.9%), the endohedral cluster also includes ^45^Sc (*I* = 7/2, 100%) and ^14^N (*I* = 1, 99.6%). 59% of carbon cages also have one or more ^13^C atoms (*I* = 1/2). Dipolar field created by nuclear spins hence limits the “intrinsic” resonance linewidth and cannot be eliminated by magnetic dilution. Finally, we could not help but notice that the very narrow resonance linewidth in diluted DySc_2_N@C_80_ corresponds to the tunneling gap *Δ*_T_ estimated above using the Landau–Zener theory. However, this correspondence should be taken with great caution in the view of the very approximate nature of the *Δ*_T_.

The discussion above was limited to the longitudinal dipolar fields. The intermolecular spin–spin interactions also create transverse fields. By acting on the transverse components of the *g*-tensor, *H*dip⊥ increases the tunneling gap and hence increases the QTM relaxation rate.^40^,^95^,^96^ Furthermore, the transverse field is necessary to open the tunneling gap in Kramers ions, and *H*dip⊥ is believed to be a crucial contribution. However, our results for DySc_2_N@C_80_ dispersed in polystyrene show that the QTM relaxation in zero field remains efficient even in the very diluted sample. Thus, we conclude that intrinsic single-molecular dipolar fields are still crucial to induce QTM.

The arguments put forward in this section are general and our conclusions are not limited to DySc_2_N@C_80_ but have broader implications. The field dependence of the QTM rates in SIMs studied for non-diluted powder samples are likely to have predominantly dipolar nature and should not be confused with an intrinsic magnetic field dependence of the QTM rate. The narrow resonance width also introduces the important question about the determination of relaxation time in strongly diluted samples by AC magnetometry. If the QTM resonance linewidth becomes very narrow, even the small sub-mT oscillation amplitude usually applied in AC measurements may partially bring the system out of the QTM resonance and hence disturb the measured time. As the conclusion on diminishing the QTM by dilution is often based on the increase of relaxation times determined by AC magnetometry, this point should be carefully examined. A decrease of the QTM drop in the magnetization curve of the diluted sample (such as seen in Fig. 3b and c) may also be misleading. If the QTM resonance is narrowed by dilution, the time when the system finds itself in resonance during the field sweep across zero field is decreased dramatically (by 1–2 orders of magnitude), leading thus to a smaller fraction of flipped spins even if the tunneling gap is not affected by the dilution.

Interestingly, in the magnetic fields exceeding the QTM threshold, diluted samples (both single crystals and powder) relax noticeably faster than the non-diluted ones (Fig. 6). The field dependence indicates that these relaxation processes can be associated with the direct relaxation mechanism, which involves phonons with the frequency, corresponding to the energy difference between the states of the opposite spins. Besides, the energy released should be further dissipated into the lattice. If the phonon density of states is low, which is the case at low temperature, the energy dissipation can be inefficient. This effect can lead to an apparent increase of relaxation time and is known as the phonon bottleneck.^91^,^97^,^98^ When spins are diluted, the phonon bottleneck is less pronounced or not relevant at all.^98^ Therefore, we propose that the difference of the relaxation times of diluted and non-diluted samples of DySc_2_N@C_80_ at high fields may be caused by the phonon bottleneck effect in the latter.

## Temperature dependence of relaxation times

**Fig. 7:**
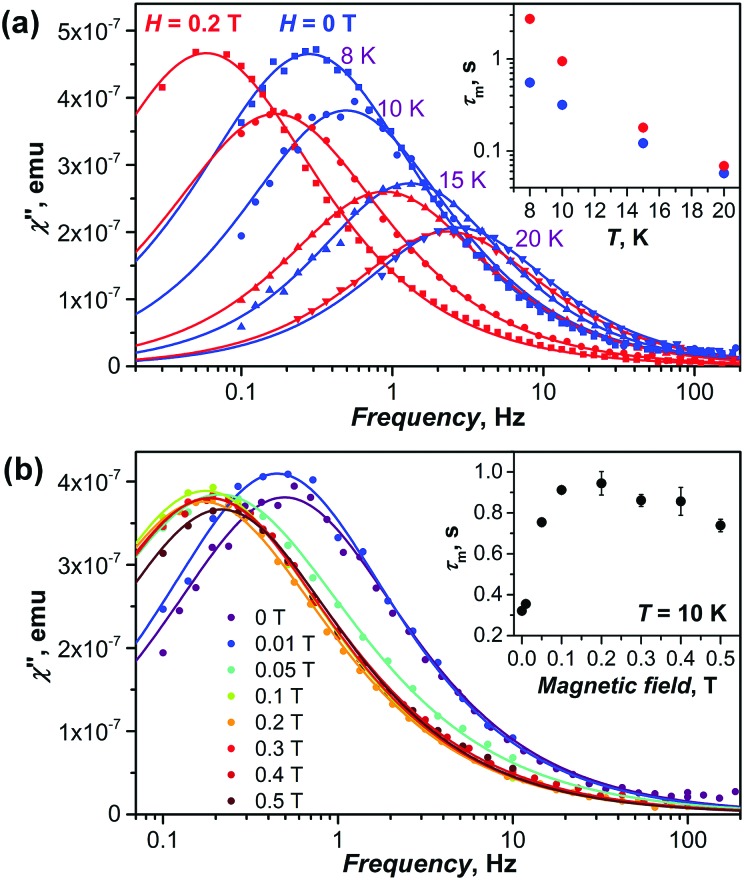
(a) Imaginary component of the magnetic susceptibility *χ*′′ of non-diluted DySc_2_N@C_80_ powder in zero field (blue dots and curves) and in the field of 0.2 T (red dots and curves) at temperatures of 8, 10, 15, and 20 K; the inset shows temperature dependence of the relaxation times. (b) Imaginary component of magnetic susceptibility *χ*′′ of DySc_2_N@C_80_ measured at 10 K in different constant field ranging from 0 T to 0.5 T; the inset shows the field dependence of relaxation times at 10 K. In both (a and b), dots are experimental *χ*′′ data points, lines are fits obtained with the generalized Debye model.

**Fig. 8:**
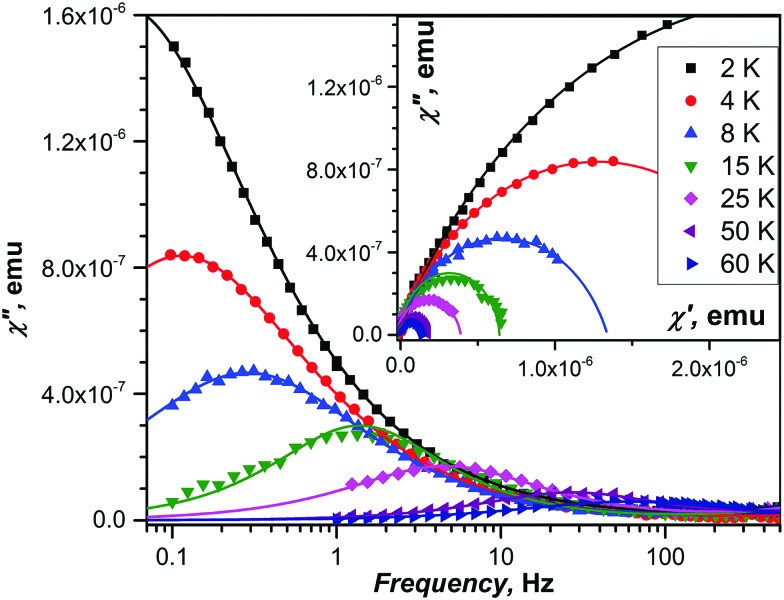
Imaginary component of the magnetic susceptibility *χ*′′ of non-diluted DySc_2_N@C_80_ powder measured in zero field at selected temperatures. The inset shows Cole–Cole plots. Dots are experimental data points, lines are fits obtained with the generalized Debye model.

DC magnetometry was used to measure relaxation times for diluted powder samples as well as for in-field relaxation of the non-diluted DySc_2_N@C_80_ at temperatures below 5 K, where relaxation times are longer than 100 s. At higher temperatures, and for zero-field relaxation of the non-diluted DySc_2_N@C_80_, determination of relaxation times requires the use of AC magnetometry (Fig. 7 and 8).

Fig. 7a shows the out-of-phase susceptibility *χ*′′ of the non-diluted DySc_2_N@C_80_ powder sample between 8 K and 20 K in zero DC field and in the DC field of 0.2 T. At 8 K, zero-field and in-field curves have distinctly different positions of their maxima, corresponding to relaxation times of 0.55 ± 0.01 and 2.72 ± 0.07 s, respectively. With further increase of the temperature, the difference between zero-field and in-field peaks is diminishing, and they become nearly indistinguishable near 20 K. A more detailed study of the field dependence of the dynamic magnetic susceptibility was then undertaken at 10 K (Fig. 7b). The *χ*′′ curve measured in the field of 0.01 T has a similar maximum position (*τ*_m_ = 0.35 ± 0.01 s) to that of the zero-field curve (*τ*_m_ = 0.32 ± 0.01 s), but is somewhat broadened towards lower frequencies. The increase of the field to 0.05 T shifts the maximum of *χ*′′ to lower frequencies, and the longest relaxation times of 0.91 ± 0.01 s and 0.94 ± 0.06 s are observed in the field of 0.1 and 0.2 T, respectively. At higher fields, a gradual decrease of the relaxation time is observed down to *τ*_m_ = 0.74 ± 0.03 s in the field of 0.5 T. The change of relaxation time with the field follows the same trend as observed by DC magnetometry at the temperature of 1.8 K (Fig. 6).

**Fig. 9:**
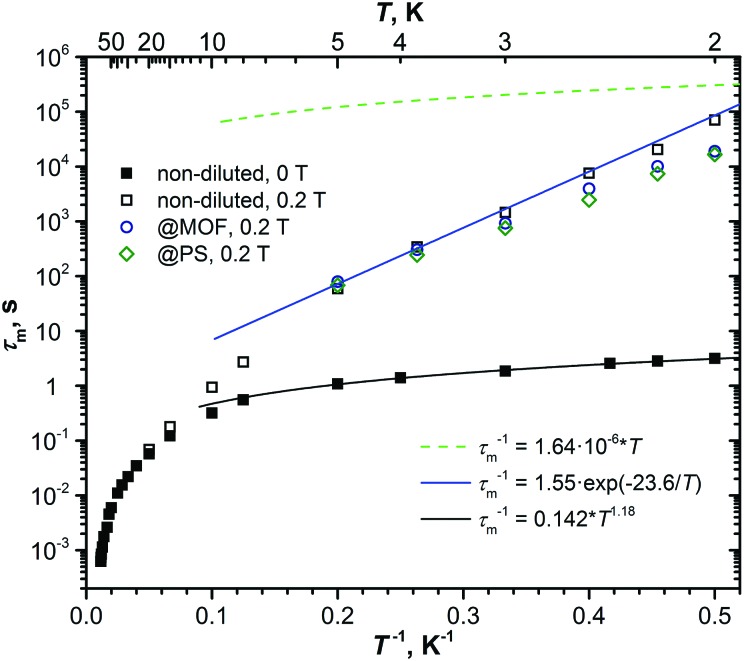
Relaxation times of magnetization of DySc_2_N@C_80_ at temperatures of 2–87 K. Zero-field values are shown as full dots, in-field (0.2 T) values are denoted as open dots. Relaxation times for non-diluted DySc_2_N@C_80_ are shown in black, the values for diluted samples are blue (dilution with MOF) and green (diluted with polystyrene, PS). The times longer or shorter than 10 s are determined by DC and AC magnetometry, respectively. The dashed green line represents the calculated rate of relaxation following the direct mechanism in the field of 0.2 T with parameters estimated from the field dependence (Fig. 6); the blue line is the fit of the points in the 2–5 K range with the Orbach relaxation mechanism. The black line represents the fit of the QTM-like zero-field relaxation with the power function of temperature.

A complete set of relaxation times measured for the non-diluted powder sample of DySc_2_N@C_80_ in the temperature range of 2–87 K is presented in Fig. 9. Temperature dependence of the magnetization relaxation rate of SMMs is usually described by a combination of Raman, Orbach, QTM, and direct processes:3
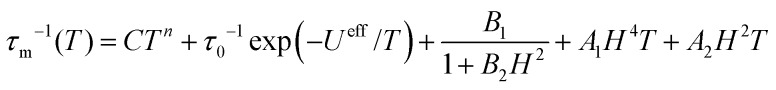
The first term in eqn (3) corresponds to the two-phonon Raman process, *C* and *n* are fitting parameters; *n* is expected to be 9 for Kramers ions in Debye theory,^91^ but in the presence of optical phonons *n* can be smaller.^99^ The second term describes the Orbach relaxation *via* an excited state with the effective barrier *U*^eff^, and the remaining terms for QTM and direct relaxation were explained above (eqn (1)). Eqn (3) has too many parameters to fit experimental data all at once, so we will analyze the temperature ranges separately trying to identify the dominant relaxation mechanism.

In zero magnetic field, SIMs usually show two temperature regimes of relaxation. The QTM dominates at low temperature and can be recognized by a temperature-independent *τ*_m_. Raman and/or Orbach relaxation processes take over at higher temperatures, leading to a fast decrease of the relaxation time. With an increase of the temperature, the Orbach mechanism should become dominant and appear as a straight line in Arrhenius coordinates, but in fact, the linear regime is not always reachable within the frequency range accessible to AC magnetometry. Finally, if a finite magnetic field is applied, the Raman/Orbach processes are not affected, whereas the QTM is switched off, leading to a much longer (sometimes several orders of magnitude) relaxation times at low temperatures.

The temperature dependence of the relaxation times of DySc_2_N@C_80_ follows the general pattern outlined above for SIMs, but with a noticeable deviation at low temperatures. Below 5 K, in-field and zero-field relaxation times are indeed different by some orders of magnitude, which would be expected for the QTM quenched by the field. But quite surprisingly, the zero-field relaxation rate shows a clear temperature dependence. Upon a temperature increase from 2 to 5 K, the relaxation time drops from 3.16 ± 0.06 to 1.08 ± 0.02 s, respectively. Fitting the relaxation rate in the 2–5 K range by a combination of QTM and a power function, *τ*_m_^–1^ = *τ*_QTM_^–1^ + *bT*^*n*^, gives a perfect match for the power function alone, with *b* = 0.142 ± 0.006 s^–1^ K^–*n*^ and *n* = 1.18 ± 0.04. An attempt to describe this temperature dependence by an Orbach process does not give a good agreement (Fig. S15, ESI†). The temperature power function with the exponent near 1 may point to a direct relaxation mechanism. But the rate of relaxation under the direct mechanism should be accelerated in finite magnetic fields (see eqn (1)), which is opposite to what is observed for DySc_2_N@C_80_. Sometimes, the direct relaxation process in the finite field is slowed down by a phonon bottleneck, which can even result in the appearance of a magnetic hysteresis.^100^–^102^ It is indeed likely that the direct process in non-diluted DySc_2_N@C_80_ is affected by the phonon bottleneck, as already pointed out above in the discussion of the field dependence at 1.8 K. However, the relaxation in diluted samples is faster only by several times, but not by five orders of magnitude. We therefore conclude that the temperature dependence of zero-field relaxation cannot be explained by a direct process. As neither Orbach nor Raman processes are field-dependent, we have to conclude that the drop of the relaxation time by almost five orders of magnitude still points to the QTM-induced relaxation in zero field. The dominance of the QTM relaxation mechanism in zero field at low temperature is also confirmed by the field dependence of relaxation times discussed in the previous sections. The origin of the temperature dependence of zero-field relaxation in the 2–5 K range thus remains unclear. We may hypothesize that although QTM itself is temperature-independent, flipping of the spins and accompanied readjustment of local dipolar fields still requires the energy dissipation *via* the lattice.^63^ If the latter is the limiting step, then the temperature dependence appears to be an indication of the phonon bottleneck.

The in-field relaxation rate at low temperature is also quite remarkable. The power function *T*^*n*^ does not describe this range well, which excludes both direct and Raman processes. Besides, parameters *A*_1_ and *A*_2_ for the direct process in eqn (3) can be determined from the field dependence of the relaxation rate shown in Fig. 6. The temperature dependence of the relaxation times according to the direct mechanism computed with these parameters is plotted in Fig. 9. As can be clearly seen, the direct process cannot describe the experimental data at *T* > 2 K as it is substantially slower. A good fit to the experimental data is obtained by an Orbach process with the *U*^eff^ of 23.6 ± 1 K and *τ*_0_ of 0.6 ± 0.2 s. The effective barrier is similar to the earlier report by Westerström *et al.*^28^ Both the size of the barrier and the prefactor are rather unusual. According to *ab initio* calculations, the Dy ion in DySc_2_N@C_80_ has a strong magnetic anisotropy and large crystal field splitting exceeding 1940 K. The first excited crystal-field state is predicted to be near 570 K. Thus, if the in-field relaxation of DySc_2_N@C_80_ at 2–5 K indeed follows the Orbach mechanism, it cannot involve excited spin states. We already observed similar Orbach processes with low-energy barriers and long attempt times in some other fullerenes (Dy_2_S@C_82_,^6^ Dy_2_@C_80_-CH_2_Ph^23^) and hypothesized that they may correspond to the relaxation *via* low-frequency vibrations of the molecules. The fact that the Raman relaxation process with the local phonon mode may be observed as an Orbach process with the barrier corresponding to the phonon frequency has been realized back in 1960s.^103^–^105^ A recent computational study of the role of phonons in spin relaxation in SMMs showed that an anharmonic phonon with finite linewidth may lead to Orbach-like behavior with the effective barrier corresponding to one half of the phonon frequency.^106^

Above 20 K, in-field and zero-field relaxation times are not distinguishable, which indicates that the relaxation mechanisms at these temperatures are field-independent, and that the QTM and direct mechanism can be excluded. Within the limitations of the magnetometer and the sample amount, we could not reach the linear regime in Arrhenius coordinates (above 87 K the AC signals are too weak to be measured reliably). Attempts to fit the whole set of relaxation times above 20 K using a combination of the Raman and Orbach mechanisms, or the Raman mechanism alone did not produce physically meaningful results and are described in ESI† (Fig. S16–S18). Linear fit of the few highest-temperature points gives a “barrier” of 550 K, but this value should be understood only as a lower bound to the real barrier, which is therefore higher. In Dy_2_ScN@C_80_ we recently found an Orbach process with *U*^eff^ of 1735 K.^29^ In good agreement with the experimental results, *ab initio* calculations showed that the most probable relaxation pathway is through the 5th excited Kramers doublet with computed energies of 1618/1641 K (two Dy ions in Dy_2_ScN@C_80_ are slightly different). For DySc_2_N@C_80_, our calculations at the same level of theory predict a similarly high barrier of 1590 K, also corresponding to the 5th ligand field excited state (Fig. S19, ESI†). The expected barrier is slightly lower than in Dy_2_ScN@C_80_, because the Dy–N bond in DySc_2_N@C_80_ is slightly longer, and the nitride ion is the main contributor to the ligand field in nitride clusterfullerenes.^7^,^8^

### Comparison of DySc_2_N@C_80_ and Dy_2_ScN@C_80_

**Fig. 10:**
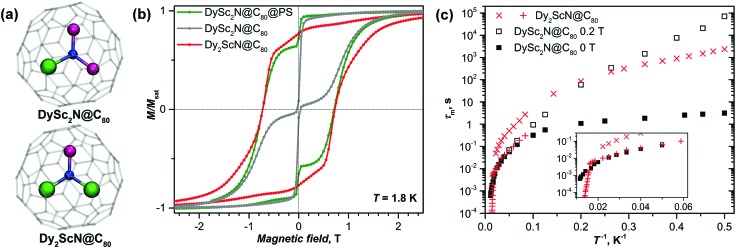
(a) Molecular structures of DySc_2_N@C_80_ and Dy_2_ScN@C_80_ (Dy is green, Sc is magenta, N is blue, carbon cage is transparent grey). (b) Magnetization curves of DySc_2_N@C_80_ (non-diluted and polystyrene-diluted powder) and Dy_2_ScN@C_80_; *T* = 1.8 K, sweep rate 2.9 mT s^–1^. (c) Relaxation times of DySc_2_N@C_80_ (zero-field and in-field non-diluted) and Dy_2_ScN@C_80_. The inset shows the enhancement of the high-temperature range.

The structure of the trimetal-nitride cluster allows for a combination of up to three lanthanide ions within one EMF molecule, and previous studies showed that DySc_2_N@C_80_, Dy_2_ScN@C_80_, and Dy_3_N@C_80_ exhibit substantially different SMM behavior at low temperatures.^16^ Dy ions in these molecules have an almost identical bonding situation (Fig. 10a), and the difference in their magnetic properties is caused by the interaction between Dy ions. Particularly illustrative is the difference between DySc_2_N@C_80_ and Dy_2_ScN@C_80_. The latter does not show fast QTM relaxation in zero field, which is explained by the ferromagnetic exchange and dipolar coupling between the magnetic moments of two Dy ions in the Dy_2_ScN cluster, thus creating an additional barrier and preventing QTM.^16^ This work on DySc_2_N@C_80_ and our recent study of Dy_2_ScN@C_80_^29^ provide comprehensive information on the relaxation times of the two EMFs in a broad temperature range and thus allow a more detailed comparison of these SMMs.

Fig. 10b shows magnetization curves of Dy_2_ScN@C_80_ and DySc_2_N@C_80_ at 1.8 K. Once the fast zero-field QTM of DySc_2_N@C_80_ is partially quenched by dilution in polystyrene, both EMFs exhibit virtually identical coercivity (0.7 T at the sweep rate of 2.9 mT s^–1^). A close similarity is also found in relaxation times (Fig. 10c). For Dy_2_ScN@C_80_, AC magnetometry revealed two relaxation processes at 12–45 K. The slower one has stronger contribution at low temperatures, and can be then further detected by DC magnetometry below 5 K. The faster process has an increased contribution at higher temperatures, and above 45 K the two relaxation pathways cannot be distinguished any more. In the 10–40 K range, the faster relaxation pathway in Dy_2_ScN@C_80_ has the same rate dependence as the relaxation of DySc_2_N@C_80_. The microscopic details of the relaxation of EMF-SMMs are not known yet, but surprisingly close relaxation times indicate that below 40 K the relaxation of magnetization in these two molecules proceeds *via* the same relaxation mechanism. However, above 45 K, relaxation in Dy_2_ScN@C_80_ is switching to the Orbach mechanism, whereas DySc_2_N@C_80_ still remains in the non-linear regime.

Before this work, Dy_2_ScN@C_80_ was believed to be the best SMM among Dy-Sc nitride clusterfullerenes for exhibiting the longest relaxation times in the broadest temperature range. However, in a finite magnetic field of 0.2 T, the relaxation times of DySc_2_N@C_80_ are more than an order of magnitude longer than those of Dy_2_ScN@C_80_ (the latter shows a field-independent relaxation rates). Above 40 K, relaxation of magnetization in Dy_2_ScN@C_80_ is considerably faster as it proceeds *via* the Orbach process with a large barrier (and hence large inclination in the temperature dependence), whereas DySc_2_N@C_80_ remains in the under-barrier regime with a less steep temperature dependence.

## Explicit modelling of magnetodynamics

During the last decade, the asymptotic eqn (3), which (aside from the QTM term) was originally developed in 1960s to describe relaxation of magnetization in paramagnetic lanthanide salts, has been very popular for the description of the relaxation of magnetization in lanthanide SMMs. Even if the true linear dependence is not observed in Arrhenius coordinates, several highest temperature relaxation points are often fitted with an Orbach regime to get an estimation of the effective barrier. More reliable but still questionable is the fitting of the non-linear part of the log(*τ*_m_)–1/*T* dependence with the Raman or a combination of the Raman and Orbach processes. Such fits often give *T*^*n*^-temperature dependences with a rather small *n*, substantially different from *n* = 9 expected from the Orbach theory for Kramers ions. The temperature dependence of the relaxation times of DySc_2_N@C_80_ discussed in the previous section is an example of the situation when eqn (3) is not able to provide a reasonable description of the relaxation rates.

**Fig. 11:**
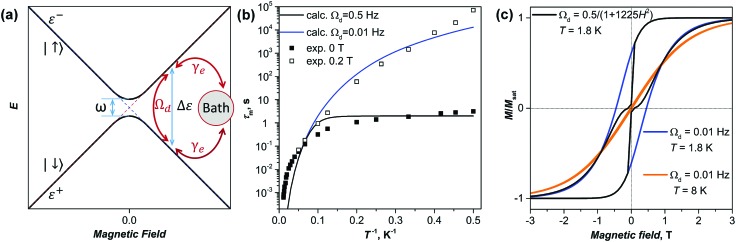
(a) Schematic description of the two-level spin system: diabatic states |↑ and |↓ have the energies of *ε*^±^ and the energy difference between the states of Δ*ε*. The avoided crossing in zero field gives the tunneling gap *ω*. Two types of dissipation pathways are denoted with red arrows: dissipation *via* the phonon bath with the temperature dependent rate *γ*_e_, and temperature-independent dissipation with the rate *Ω*_d_. (b) Comparison of computed and experimental temperature dependences of relaxation times, computations are performed with two different values of *Ω*_d_ and the empirical mean-field *γ*_e_ parameter defined as *γ*_e_ ∼ *T*^6^. (c) Magnetic hysteresis curves computed with at 1.8 and 8 K for a constant parameter *Ω*_d_ = 0.01 Hz, and computed at 1.8 K for the field dependent parameter *Ω*_d_ = 0.5/(1 + 1225*H*^2^).

The limited insight into the actual spin–lattice relaxation mechanism governing the relaxation of magnetization in SMMs at intermediate temperatures provided by the Raman term in eqn (3), and above all the lack of an obvious connection with microscopic molecular parameters of the SMMs emphasize the need of the microscopic approaches involving the true spin dynamics. More realistic descriptions of the relaxation in molecular magnets have been obtained recently with the use of master equations for spin-density propagation involving *ab initio* derived transition probabilities and spin–phonon coupling parameters,^106^,^107^ and the pitfalls of using the phenomenological description have been pointed out. Here we show that even a relatively simple model based on the two-level system and a minimal set of parameters, but involving true spin dynamics with dissipative Lindblad term can predict hysteresis and magnetization relaxation curves similar to those observed experimentally for DySc_2_N@C_80_ (Fig. 11).

The standard technique to approach the quantum dynamic problem is to solve the Liouville–von Neumann equation of motion:^108^,^109^
4
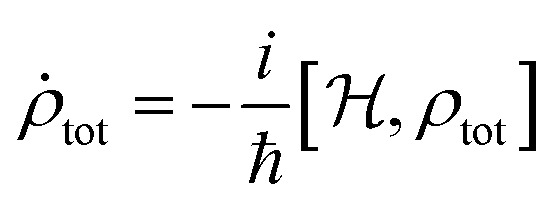
where *ρ*_tot_ is the total density operator, which includes all degrees of freedom of the system and environment in an intertwined way. Having traced out the environmental degrees of freedom in eqn (4), one would obtain the system density matrix *ρ* = Tr_env_[*ρ*_tot_].^108^,^110^ The common trace-preserving and completely positive form of this evolution is the Gorini–Kossakowski–Sudarshan–Lindblad (GKSL) equation, with dissipative Lindblad term 
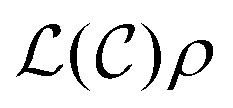
 and a collapse operator 
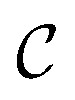
:^111^,^112^
5
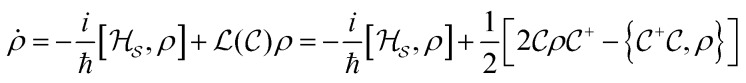
Here, eqn (5) assumes only one collapse operator 
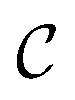
 but if necessary, more 
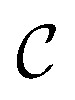
-operators and corresponding 
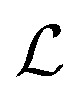
-terms can be introduced depending on the nature of the system.

Deep mathematical elaboration goes beyond the scope of this report and will be reported elsewhere. It is important to admit though, that in such a formulation, we have the Hermitian operator 
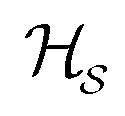
, which governs the systems dynamics, with a separate construction built upon the dissipation operator 
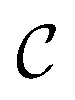
. The latter itself has the inner structure 
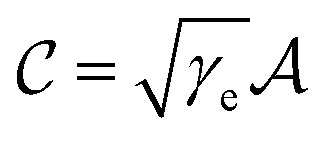
, which combines the environment coupling operator 
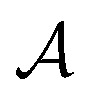
 and the interaction scaling parameter *γ*_e_, which has the physical meaning of a rate. To this end, the problem is reduced to a proper definition of 
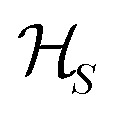
 and 
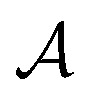
 operating in the proper space, and the coupling rates. Importantly, the GKSL approach provides a phenomenological description of the systems. In many cases the collapse operators can be guessed or be physically justified. For those problems the GKSL is definitely the method of choice. However, when the physical insight is not sufficient to construct a system-environment interaction operator 
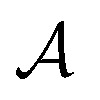
, the first-principle methods must be employed to compute the energy spectra and couplings with the environment.^30^,^106^ Here, we use the GKSL method, as stable and always leading to physically relevant density matrixes, given the collapse operator is physically justifiable.

The two-level quantum system considered here (Fig. 11a) with the states |↑ and |↓ is isomorphic to a spin 1/2 system, which can be either real spin or a pseudospin. The spin inversion operator is then defined as *σ̂*_*z*_ = |↓↓| – |↑↑|, whereas raising and lowering operators *σ̂*_+_ and *σ̂*_–_ are |↑↓| and |↓↑|, respectively. The energy splitting between the states Δ*ε* as a function of the magnetic field *H* depends on the spin *g*-factor, and in the case of DySc_2_N@C_80_ (pseudospin with *g*_*z*_ of 20) Δ*ε*(*H*) amounts to 0.2 THz per Tesla. The coupling between the diabatic states |↑ and |↓ is *ω*. For Dy^3+^ in DySc_2_N@C_80_ with a strongly axial ligand field, *ω* is very small and is estimated from CASSCF/RASSI calculation to be 10^–4^–10^–5^ Hz for the ground Kramers doublet. In these terms, the system Hamiltonian leads to the master equation:6
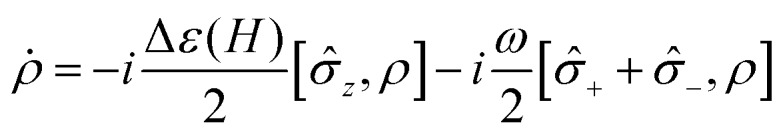
To address dissipation, a collapse operator 
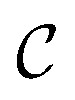
 should be introduced then. In the simple settings of the two-level system the temperature effects are given by *γ*_e_ = *γ*_e_(*T*) (explicit phonons can be also introduced into the system Hamiltonian, but this elaboration will not be discussed here). To account for the magnetic field dependence of the magnetization 
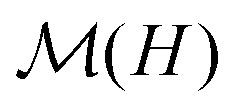
, the structure of the collapse operator itself should guide the system onto a correct steady state value at infinite time. The thermodynamic limit of populations of states is governed by Boltzmann statistics, giving the temperature-dependent collapse operator of the following form:7
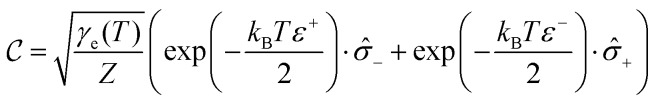
here *Z* is the two-state partition function and *ε*^±^(*H*) = ±*gμ*_B_*H* are energies of the states |↑ and |↓ as a function of field. Substitution of this collapse operator into eqn (5) allows recovery of the thermodynamic limit (*t* → ∞) at any *H* or *T* (see ESI† for details).

The operator in eqn (7) does not include temperature independent relaxation, such as zero-field QTM observed for DySc_2_N@C_80_ and many other Dy-SIMs. Within the proposed framework this process can be easily introduced through a temperature independent relaxation channel by an additional collapse term 
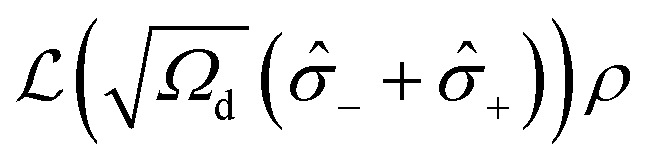
, giving the total master equation:8
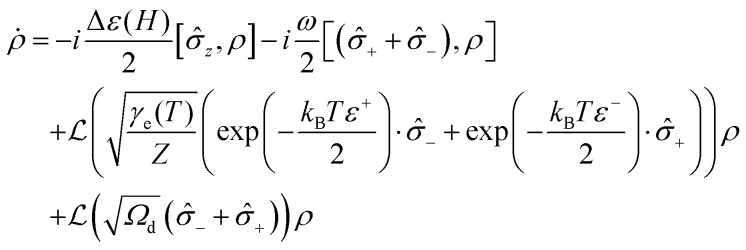
Quantum spin dynamics according to eqn (8) requires the definition of two control parameters, the spin–phonon coupling *γ*_e_(*T*) and the temperature-independent rate *Ω*_d_ of collapse onto the *M*(*t*)_*δ*_ = 0 state. In this work we use a single mean-field temperate-dependent parameter defined as a power function of temperature, *γ*_e_(*T*) ∼ *T*^6^, where the exponent 6 is chosen arbitrarily to provide an agreement with the experimental relaxation times in the broad temperature range. When a complete Hamiltonian for the magnetic center is built with explicit definition of phonons, the spin–phonon coupling and its temperature dependence in *γ*_e_ can be calculated in a linear response limit as in ref. 30, 106 and 107. The ability of the system to show QTM-like relaxation behavior is controlled by the parameter *Ω*_d_. Fig. 11b shows results of the relaxation curves modeling^113^,^114^ with *Ω*_d_ = 0.5 Hz, which resembles the relaxation behavior of the non-diluted powder DySc_2_N@C_80_ with QTM in zero field, and for the system with the reduced value of *Ω*_d_ = 0.01 Hz, which gives the temperature dependence of the relaxation time similar to that observed for the powder sample of DySc_2_N@C_80_ in the field of 0.2 T, when the QTM is not efficient.

Fig. 11c shows hysteresis curves predicted using the same parameters as above and assuming field sweeping axioms discussed in detail in ESI.† When *Ω*_d_ is small, 0.01 Hz, the magnetic hysteresis with remanence is predicted at 1.8 K, whereas at 8 K the hysteresis is found to be almost closed for the same simulated sweep rate. To properly include the QTM in the modelling of magnetic hysteresis, the value of *Ω*_d_ should be field-dependent to reflect the decrease of the coupling between the states |↑ and |↓ with the increase of the magnetic field. The dependence *Ω*_d_ = 0.5/(1 + 1225*H*^2^), which recovers the *Ω*_d_ values of 0.5 Hz in zero field and 0.01 Hz in the field of 0.2 T, gives a butterfly-shaped hysteresis curve as observed experimentally for the systems with zero-field QTM.

It cannot be expected that the two-level system with a single mean-field temperature-dependent dissipation parameter can describe a real system in all its complexity. Nevertheless, this simple model can grasp the general details of the relaxation, making it a convenient framework for a more comprehensive elaboration on the relaxation dynamics in future. It is relatively straightforward to add microscopic parameters of the spin–phonon coupling from *ab initio* calculations into the temperature-dependent *γ*_e_(*T*) term or increase the number of the spin states involved in the consideration, but this modelling goes beyond of scope of this work and will be reported elsewhere.

## Conclusions

In this work we presented comprehensive studies of the relaxation of magnetization in the archetypical fullerene-based single ion magnet, DySc_2_N@C_80_, in the form of powder and single crystals. Dilution of the compound in three diamagnetic matrices, diamagnetic fullerene, metal–organic framework, and a polymer, was studied and resulted in a noticeable change of the magnetic hysteresis curves at low temperature. The study of the field dependence of the relaxation rate near zero magnetic field, where the molecule shows fast QTM relaxation, revealed a strong narrowing of the QTM resonance, from 150 mT in the non-diluted powder to less than 1 mT in the DySc_2_N@C_80_ diluted in a large excess of polystyrene. The narrowing was found to correlate with the variation of the intermolecular dipole–dipole interactions of Dy^3+^ spins. At the same time, rather efficient zero-field QTM observed in strongly diluted samples indicates that intermolecular interactions do not play a crucial role in the opening of the tunneling gap in the Dy^3+^ spin levels in DySc_2_N@C_80_. This result shows that great care is needed for determination of zero-field relaxation times in strongly diluted samples as it is easy to either miss the resonance field position in DC measurement or to drive the system out of resonance in AC measurements.

The study of the temperature dependence of the relaxation rates showed a surprising phenomenon, a weak temperature dependence of the relaxation rate proceeding under QTM mechanism at 2–5 K. As the QTM nature of the zero-field relaxation is beyond any doubts, the nature of the temperature dependence remains unclear and may be tentatively explained by slow energy dissipation in the lattice, similar to the phonon-bottleneck effect. In-field relaxation in the 2–5 K range is best described by the Orbach mechanism with the effective barrier of 23.6 ± 1 K. As this value is at least an order of magnitude smaller than the lowest-energy ligand-field excited state predicted by *ab initio* calculations, we hypothesize that the barrier corresponds to the local phonon strongly coupled to the spin system. At temperatures above 20 K all field dependence of the relaxation rate vanishes. The relaxation in this regime is usually described by Raman and/or Orbach mechanisms. We could not reach linear dependence in Arrhenius coordinates up to 87 K. At the same time, the power function of temperature expected for the Raman mechanism also does not provide a reasonable description of the relaxation rates in this temperature range. These results clearly point to the limitations of the traditional asymptotic description developed for relaxation of magnetization in paramagnetic salts in the 1960s and emphasize the need for approaches relying on microscopic parameters of individual SMM molecules. The framework for the study of the true spin dynamics in SMMs based on the system Hamiltonian with dissipative Lindblad term is presented.

## Conflicts of interest

There are no conflicts to declare.

## Supplementary Material

Supplementary information

Crystal structure data
