# Supplementary material for: Magnetization relaxation in the single-ion magnet DySc2N@C80: quantum tunneling, magnetic dilution, and unconventional temperature dependence
Source: Phys Chem Chem Phys. 2018 Apr 10;20(17):11656–72. doi: 10.1039/c8cp01608a (PMC5933001; doi:10.1039/c8cp01608a)
Supplement: Supplementary file 1 [file CP-020-C8CP01608A-s001.pdf]

Electronic Supplementary Material (ESI) for Physical Chemistry Chemical Physics.
This journal is © the Owner Societies 2018

Magnetization relaxation in the single-ion magnet $DySc_2N@C_{80}$: quantum tunneling, magnetic dilution, and unconventional temperature dependence

D. S. Krylov,[1] F. Liu,*[1] A. Brandenburg,[1] L. Spree,[1] V. Bon,[2] S. Kaskel,[2] A. U. B. Wolter,[1] B. Büchner,[1] S. M. Avdoshenko,*[1] A. A. Popov*[1]

Supporting information

**Synthesis and separation of $DySc_2N@C_{80}$**	**S2**
**X-ray crystallographic data**	**S4**
**Adsorption of $DySc_2N@C_{80}$ by the MOF DUT-51(Zr)**	**S8**
**Dilution in polystyrene**	**S10**
**Magnetic measurements of the single crystal**	**S11**
**Determination or magnetic relaxation times with DC magnetometry**	**S13**
**Magnetic relaxation times determined by DC magnetometry**	**S20**
**Magnetic relaxation times from AC magnetometry**	**S23**
**Fitting of magnetic relaxation times with different mechanisms**	**S24**
**Ab initio calculations for $DySc_2N@C_{80}$**	**S28**
**Simulation of magnetic hysteresis curves from spin dynamics**	**S31**
**References**	**S34**

Synthesis and separation of $DySc_2N@C_{80}$

$DySc_2N@C_{80}$ was synthesized in a modified Krätschmer-Huffman fullerene generator by vaporizing composite graphite rods (ϕ 6 × 100 mm) containing a mixture of Dy_2O_3, Sc_2O_3, guanidine thiocyanate (GT, as solid nitrogen source[1]) and graphite powder with the addition of 180 mbar He as described previously.[2] The as-produced soot was Soxhlet-extracted by CS_2 for 24 h, and the resulting brown-yellow solution was distilled to remove CS_2 and then immediately re-dissolved in toluene and subsequently passed through a 0.2 μm Telflon filter for HPLC separation. The isolation of $DySc_2N@C_{80}$ was performed by multi-step high performance liquid chromatography (HPLC). The purities of the isolated $DySc_2N@C_{80}$ was further checked by laser desorption/ionization time-of-flight (LDI-TOF) mass spectroscopic (MS) analysis.

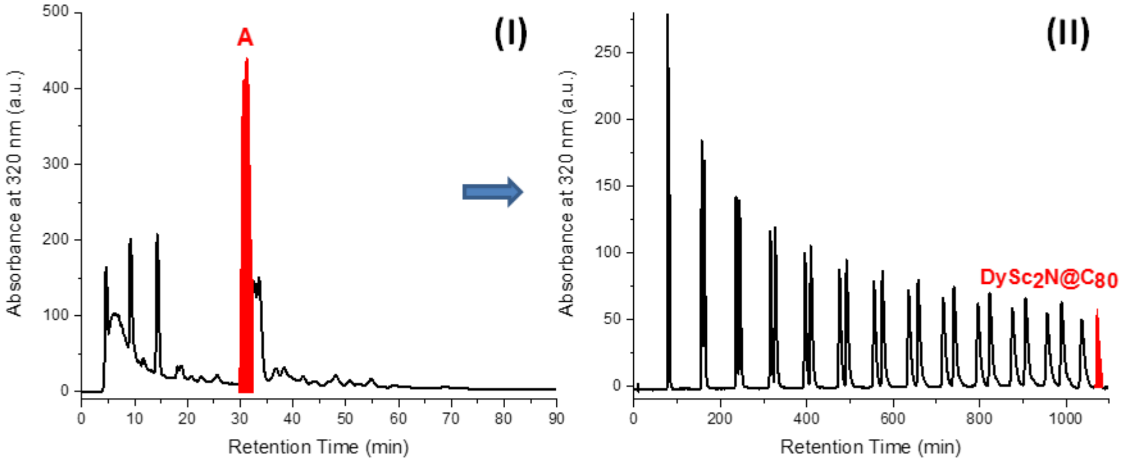

Figure S1. Separation of $DySc_2N@C_{80}$. Two steps were required to obtain pure $DySc_2N@C_{80}$ from the extracted fullerene mixture. **(I)** HPLC profile of the fullerene mixture extracted from Dy_2O_3-Sc_2O_3- GT. Fraction A contains the target $DySc_2N@C_{80}$. (HPLC conditions: linear combination of two 4.6 × 250 mm Buckyprep columns; flow rate 1.6 mL/min; injection volume 800 μL; toluene as eluent; 40 °C) **(II)** Recycling HPLC profile of fraction A (10 × 250 mm Buckyprep column; flow rate 2 mL/min; injection volume 4.5 mL; toluene as eluent). Pure $DySc_2N@C_{80}$ was obtained.

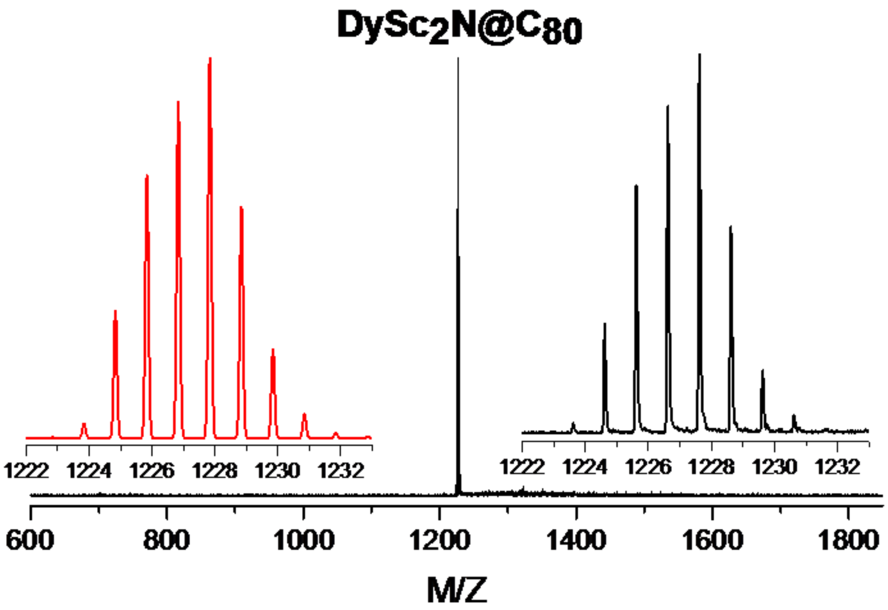

Figure S2. Positive-ion laser desorption/ionization time-of flight (LDI-TOF) mass spectrum of the purified $DySc_2N@C_{80}$. Insets: measured (black) and calculated (red) isotopic distributions of $DySc_2N@C_{80}$.

X-ray crystallographic data for single crystals of $DySc_2N@C_{80}$

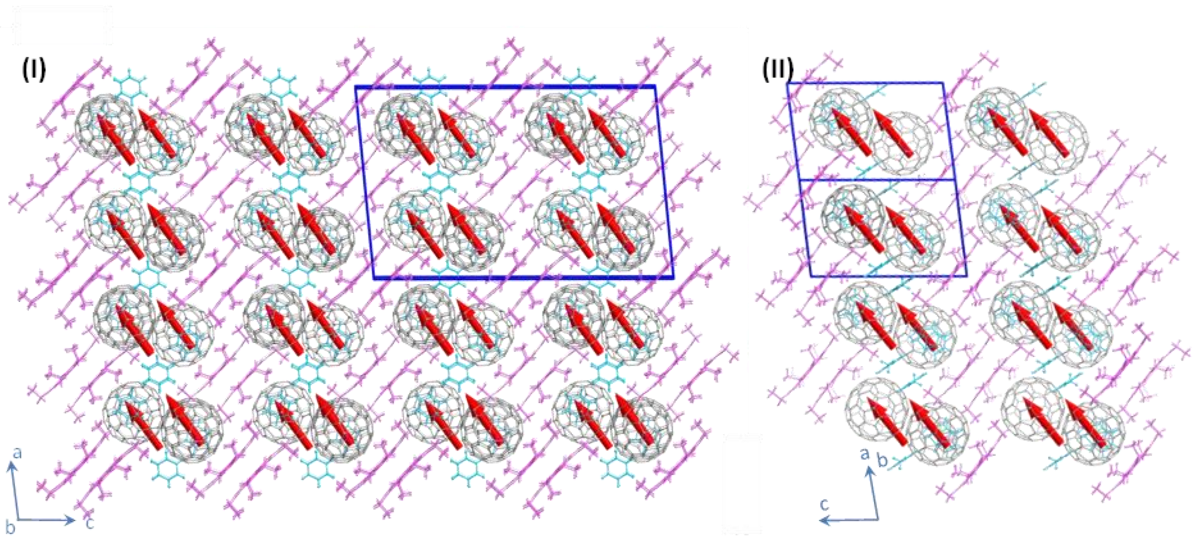

Figure S3. Comparison between the packing mode of $DySc_2N@C_{80}$ in crystals **I** (left) and **II** (right). The atoms of the C_{80} cage is shown with pale grey, the atoms of the solvents are shown with sky blue, while the atoms of the Ni(OEP) are shown with pale pink. The Dy1A-N1 bond directions are highlighted with arrows sit on the Dy1A atom.

X-ray crystallographic data on $DySc_2N@C_{80}/Lu_3N@C_{80}$ (1:9)

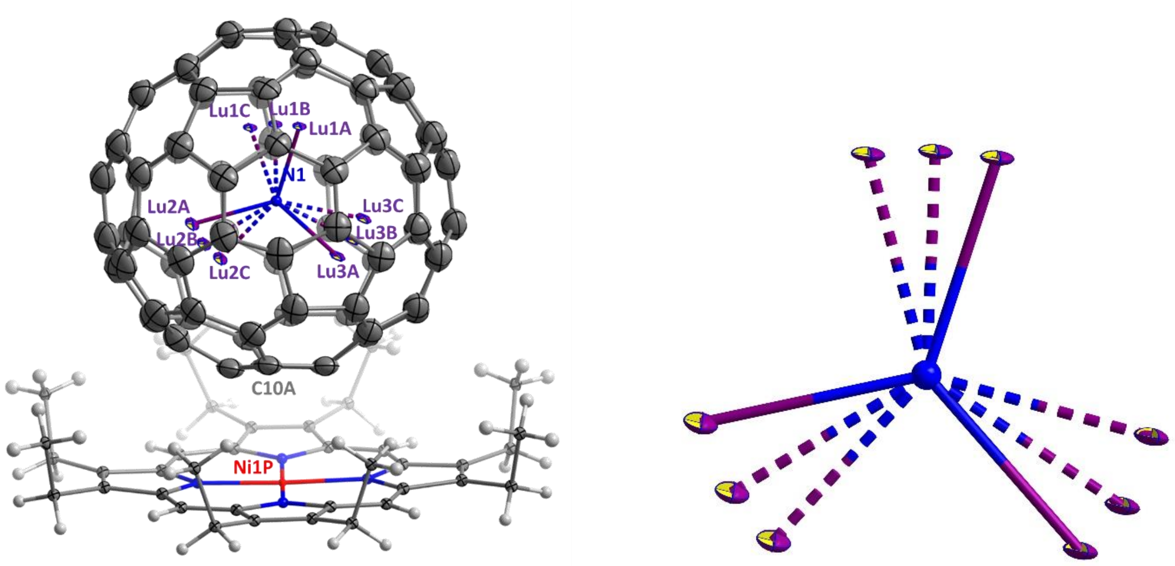

Figure S4. Mutual orientation of the $Lu_3N@C_{80}$ and NiOEP molecules (fullerene ellipsoids are shown at the 20% probability level, Ni1P - C10A distance is 2.833 (0.022) Å.

Table S1. Crystal data and data collection parameters.

Crystal	$DySc_2N@I_h(7)$-C_{80} · $Ni^{II}(OEP)$ · $2(C_6H_6)$	$DySc_2N@I_h(7)$-C_{80} · $Ni^{II}(OEP)$ · $0.72(C_6H_6)$ · $1.28(C_7H_8)$	$Lu_3N@I_h(7)$-C_{80} :$DySc_2N@I_h(7)$-C_{80}=10 · $Ni^{II}(OEP)$ · $2(C_6H_6)$
Formula	C128 H56 Dy N5 Ni Sc2	C129.29 H58.28 Dy N5 Ni Sc2	C128 H56 Lu2.60 N5 Ni
Formula weight	1935.85	1992.64	2177.41
Color, habit	Black, block	Black, block	Black, block
Crystal system	monoclinic	triclinic	triclinic
Space group	$C2/c$	$P\bar{1}$	$P\bar{1}$
a, Å	25.190(5)	14.570(3)	14.683(3)
b, Å	15.050(3)	14.680(3)	14.720(3)
c, Å	39.490(8)	20.280(4)	19.750(4)
α, deg	90	83.73(3)	85.27(3)
β, deg	95.46(3)	84.16(3)	85.50(3)
γ, deg	90	60.60(3)	61.94(3)
Volume, Å^3	14903(5)	3750.6(16)	3750.1(16)
Z	8	2	2
T, K	100	100	100
Radiation (λ, Å)	Synchrotron Radiation (0.89429)	Synchrotron Radiation (0.89429)	Synchrotron Radiation (0.89429)
Unique data (R_{int})	16413 (0.0930)	12527 (0.0444)	8849 (0.1017)
Parameters	1239	1219	1202
Restraints	1210	1038	1936
Observed data ($I > 2\sigma(I)$)	9234	12023	13727
$R_1{}^a$ (observed data)	0.1131	0.1548	0.1787
$wR_2{}^b$ (all data)	0.3667	0.4295	0.4919
CCDC NO.	1587626	1587784	-

^{a}For data with $I > 2\sigma(I)$, $R_1 = \frac{\sum||F_o|-|F_c||}{\sum|F_o|}$. ^{b}For all data, $wR_2 = \sqrt{\frac{\sum\left[w\left(F_o^2-F_c^2\right)^2\right]}{\sum\left[w\left(F_o^2\right)^2\right]}}$

Table S2. The selected Dy-N bond length table.

Dy-N bond environment	Dy-N (Å)	reference
Tertiary amines (NR_3)	2.602(31)	[3]
Hydrazines (σ-NR_2NR_2)	2.570	[3]
N-Isothiocyanates	2.433	[3]
Guanidinoacetaminates	2.178(8), 2.506(7), 2.395(8), 2.272(8)	[4]
$Dy_2(C_5H_5)_3(NPPh_3)_3$	2.154(7), 2.318(7), 2.338(7)	[5]
(Ferrocenediyl($NHSitBuMe_2)_2$)DyI$(THF)_2$	2.21(2), 2.20(6)	[6]
N_1,N_3-bis(3-methoxysalicylidene) diethylenetriamine ligand coordinated Dy compound	2.14(2), 2.54(3), 2.228(10), 2.53(2), 2.26(2), 2.74(2)	[7]
$Dy[N(SiMe_3)_2]_3$	2.183(4), 2.213(3), 2.257(3)	[8]

Adsorption of $DySc_2N@C_{80}$ by the MOF DUT-51(Zr)

$DySc_2N@C_{80}$ in toluene was prepared for the DUT-51(Zr) adsorption experiment. The amount of the $DySc_2N@C_{80}$ was estimated with UV-vis-NIR absorption spectroscopy according to the Lambert-Beer's Law: $A=\lg(I_0/I)=L\cdot c\cdot \varepsilon$, where A is absorbance, L is the optical length (1 cm), C is concentration, and ε is molar extinction coefficient at 420 nm, 15.8 x 10^3 $M^{-1}cm^{-1}$, taken from the value of $Sc_3N@C_{80}$ based on their similar structure.[9] The concentration of $DySc_2N@C_{80}$ during the adsorption was monitored by UV-vis-NIR spectroscopy. The results are shown in Figure S5. After a fast drop of the fullerene concentration in solution caused by adsorption into the voids of DUT-51(Zr) during the first 24 hours, the steady concentration was achieved after ca 100 hours, as shown in Figure S6. The fullerene solution over the DUT-51(Zr) was refreshed four times reaching in the end the fullerene/DUT-51(Zr) mass ratio of ca. 1:100 (0.18 mg of $DySc_2N@C_{80}$ was adsorbed by 19.6 mg DUT-51(Zr)).

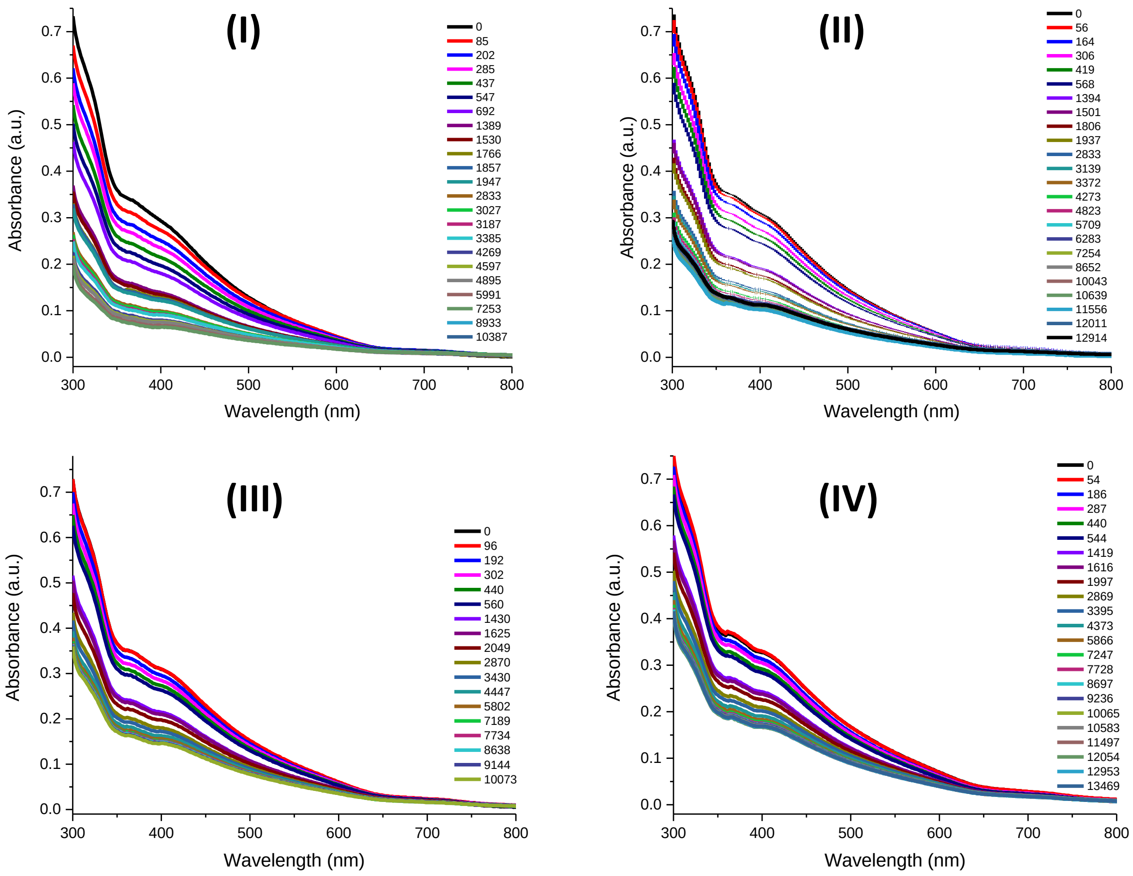

Figure S5. The UV-vis-NIR spectra of the $DySc_2N@C_{80}$ solution monitored during the adsorption by DUT-51(Zr). (I) The first adsorption. (II) A fresh $DySc_2N@C_{80}$ solution was added for the second adsorption after the first adsorption finished; so as to (III) and (IV).

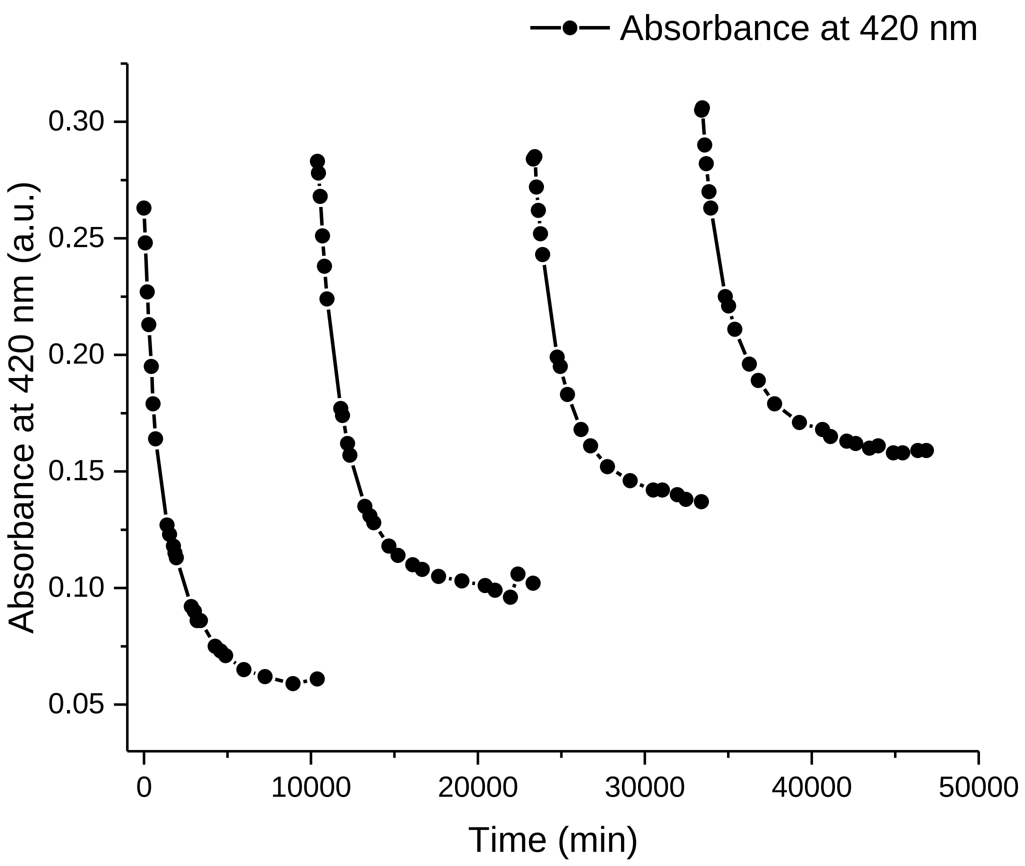

Figure S6. The concentration evolution of $DySc_2N@C_{80}$ for the adsorption presented with the absorption of the solution at 420 nm. The data was extracted from Figure S5.

Dilution in polystyrene

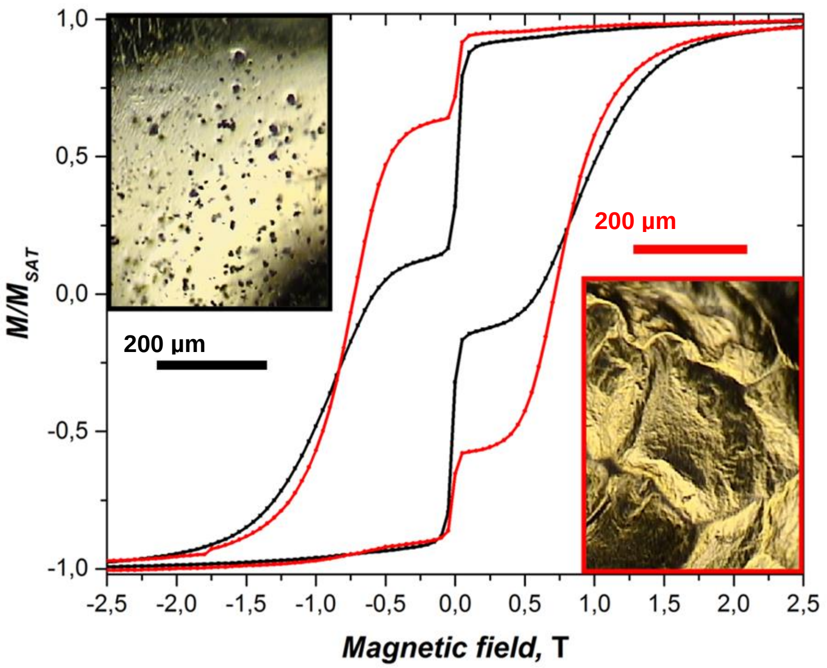

Figure S7. Magnetization curves (T = 1.8 K) and microscope images of $DySc_2N@C_{80}$ diluted with polystyrene and drop casted from different solvents: toluene (black), CS_2 (red). Crystals of fullerene can be clearly seen in the film drop-casted from toluene, whereas the film obtained from CS_2 is much more homogeneous. Homogeneity of the has a strong influence on magnetic hysteresis.

Magnetic measurements of the single crystal

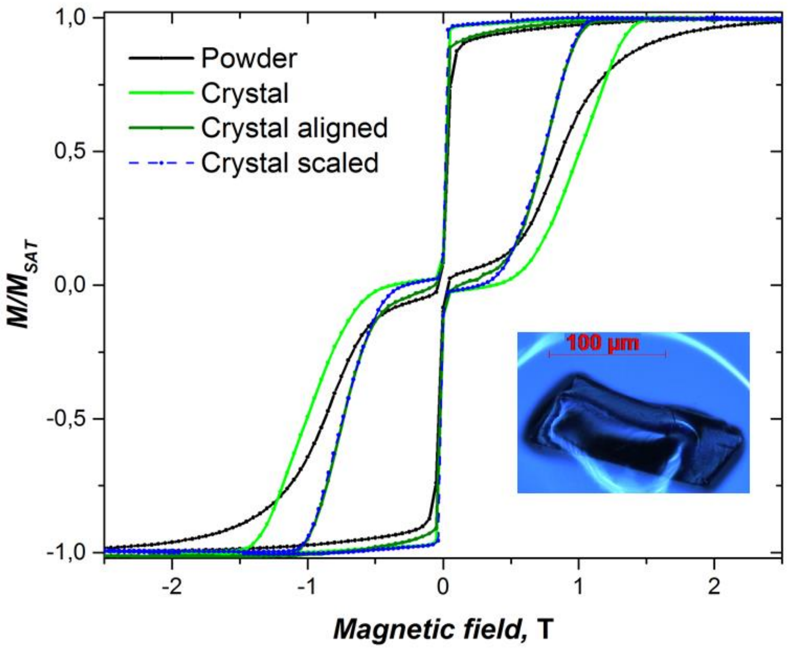

Figure S8. Normalized magnetization curves of $DySc_2N@C_{80}$ powder (black) and single-crystal of $DySc_2N@C_{80}\cdot Ni^{II}(OEP)$. Initially the crystal was aligned with the long side of the block along the direction of the magnetic magnetic field. Light green curve is the magnetic curve for this orientation of the crystal (cooled-down in zero field). When the crystal is cooled down in the presence of magnetic field, it rotated by ca 45°. Magnetization curve of the magnetically aligned crystal is shown in olive. Also shown for comparison is the magnetization curve for the non-aligned crystal, which magnetic field axis is scaled by a factor of 0.73, which is equivalent to the rotation of the magnetic field direction by 43°. The perfect match between magnetization curves of the magnetically-aligned crystal and of the scaled curve of the non-aligned crystal can be seen.

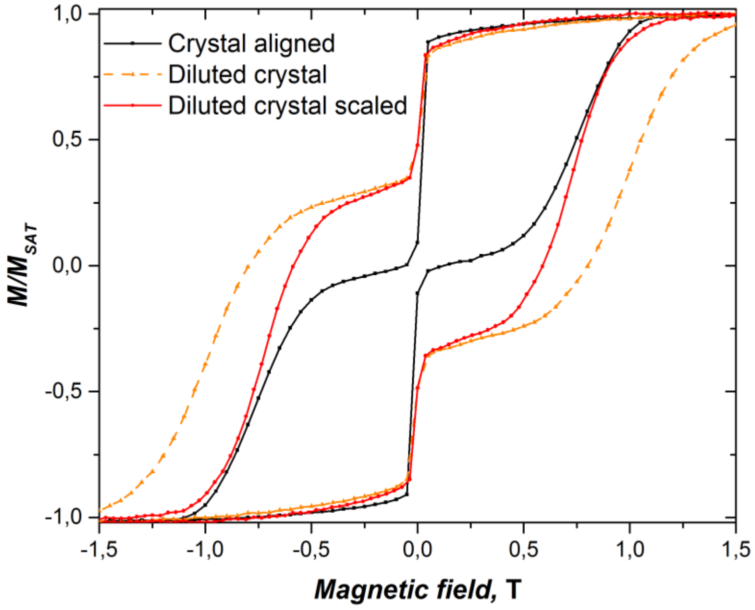

Figure S9. Magnetization curves of the magnetically aligned non-diluted single crystal (black) and diluted single-crystal (orange dashed) of $DySc_2N@C_{80}·Ni^{II}(OEP)$. The red curve is obtained from magnetization curve of the diluted crystal by scaling the magnetic field with a factor of 0.73 (equivalent to the rotation of the magnetic field by an angle 43°).

Determination or magnetic relaxation times with DC magnetometry

The two most common macroscopic approaches for determination of the system's characteristic times are: DC relaxation measurements (above 100 s, relaxation curves are recorded) and AC relaxation measurements (below 10 s, searching for the resonance behavior of the sample). The DC measurement of the magnetization decay curve is relatively simple and doesn't require large sample amounts (5-10 % compared to AC). However, difficulties arise during fitting of the experimental data as single-molecule magnets tend to exhibit a time-dependent decay rate. One of the reasons is arising from the evolution of internal dipolar fields in the sample during relaxation. Consequently a single exponential function often fails to describe the system's behavior. In rare cases double exponential description is sufficient [10]. However, in a general case the decay curve consists of an infinite number of exponentials. A characteristic value for the relaxation time distributions has to be derived. It becomes possible with a stretched exponential:

$$f(t) = M_{eq} + (M_0 - M_{eq})\, exp\left[-\left(\frac{t}{\tau_1}\right)^{\beta}\right] \tag{S.1}$$

Where M_{eq} and M_0 are the equilibrium and initial magnetizations, respectively, τ_1 is a characteristic relaxation time and β is an additional parameter that corresponds to the time-dependent decay rate $\tau^{-1} \sim t^{\beta-1}$ with $\beta = (0;1)$. In the extreme case of $\beta = 1$ one obtains a single exponential.

The stretched exponential can be represented in the form that corresponds to the continuous sum of exponential decays:

$$exp\left[-\left(\frac{t}{\tau_1}\right)^{\beta}\right] = \int_0^{\infty} P(s,\beta)\exp\left(-s\frac{t}{\tau_1}\right)ds \tag{S.2}$$

Where $P(s,\beta)$ is a normalized probability distribution [11].

For the first time the stretched exponential was proposed by Rudolf Kohlrausch in 1854 to describe the relaxation of charge from a glass Leyden jar (the precursor of modern capacitors). This function is commonly used in the studies of glassy materials, were heterogenic processes are expected. For example, the stretched exponential relaxation can be found in simulations of the 3D spin glass with large enough number of spins [12]. In the field of single-molecule magnets a time-dependent decay rate was found already for Mn_{12}ac, the first molecular magnet, consequently a stretched exponential was used [13]. The detailed comparison of the most common characteristic time derivation strategies is presented below.

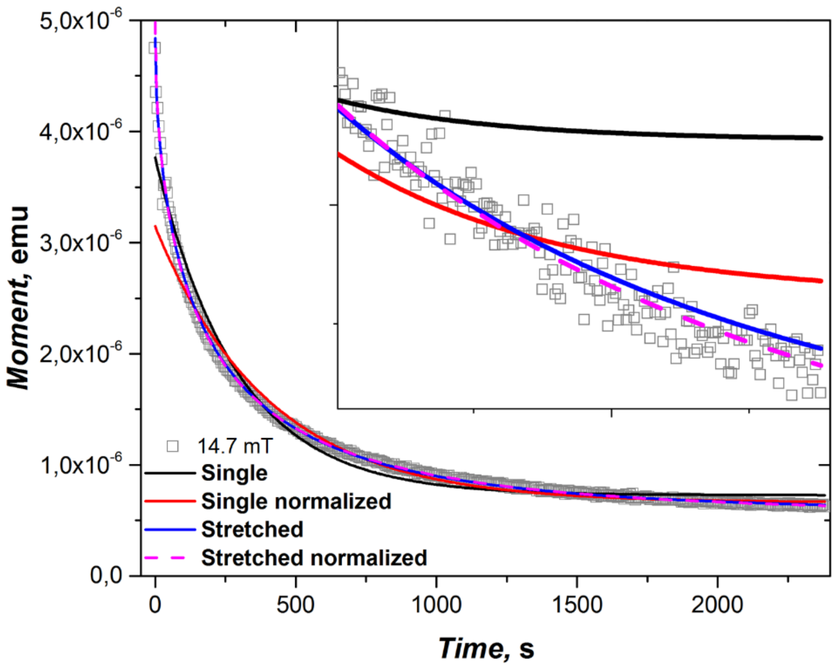

Figure S10. An example of a magnetization decay curve with a relatively fast relaxation time of 179 seconds ($DySc_2N@C_{80}$, single crystal, 1.8 K, 14.7 mT). The inset shows the final part of the relaxation curve. The large deviation of the single exponential fits indicates a time-dependent decay rate.

The two extreme cases of relaxation experiments should be taken into account. The first one corresponds to short relaxation times, which are close to the limit of the DC measurement technique. The limitation arises from the characteristics for the superconducting magnet, used in the experiment. Prior to recording the relaxation curve one has to magnetize the sample in the saturation field and then sweep the field down and stabilize in at the desired value. The common change of the magnetic is about 2-3 Tesla. For the modern QD MPMS3 magnetometer the delay between the start of sweeping and the first measured point is between 15 and 30 seconds. Thus, samples with fast relaxation undergo a considerable demagnetization already before the actual measurement starts.

The consequences are different for a constant and a time-dependent decay rates. While for the first case a single exponential fitting of the whole decay curve or a part of it would provide the same result, for the second case a curve that misses the initial part would provide overestimated values due to a biased representation of the real time distribution. Considerable deviations are found for the times below 100 s (QD MPMS3), therefore such results are excluded.

On Figure S10 an example of a relatively fast decay is shown. For such cases the measurement over more than 10 characteristic times is affordable, which provides an easier estimation of the equilibrium magnetization. This is crucial for the correct fit as the result exhibits a strong dependence on the value of M_{eq}. As a consequence the fitting algorithm has to be chosen carefully. By default most of the routines are dealing with minimization of the sum of squared residuals (the differences between a data points and the

corresponding model estimates). This leads to unequal treatment of the "first" and the "last" points of the curves, as their values can differ by several order of magnitude, still having the same instrumental error. To ensure that the average residual can't surpass the small values of the "last" points, the "normalized" residuals have to be introduced, calculated as the ratio between the residual and the corresponding value. Effectively this forces the fitting curve to follow the tail of the experimental curve. According to Figure S10 the stretched exponential describes the magnetization decay in $DySc_2N@C_{80}$ single crystal significantly better than a single exponential, and the "normalized" residuals approach increases the fit quality for the last points.

An alternative way of treating the time-dependent relaxation rate can be found in literature. The evolution of internal dipolar fields in the sample during relaxation can lead to a square-root time dependence of the signal [14]. However, this special type of relaxation is expected only in the beginning of the experimental curve, as the effect of dipolar fields vanishes towards the equilibration of the system. On Figure S11 the relaxation curves for $DySc_2N@C_{80}$ single crystal are shown. In the coordinates ued the square-root time dependence should appear as straight line, and one can see that only few first points on the experimental curve can be tentatively attributed to a square-root time dependence. Consequently, this specific regime in $DySc_2N@C_{80}$ is not accessible by the available magnetometry techniques.

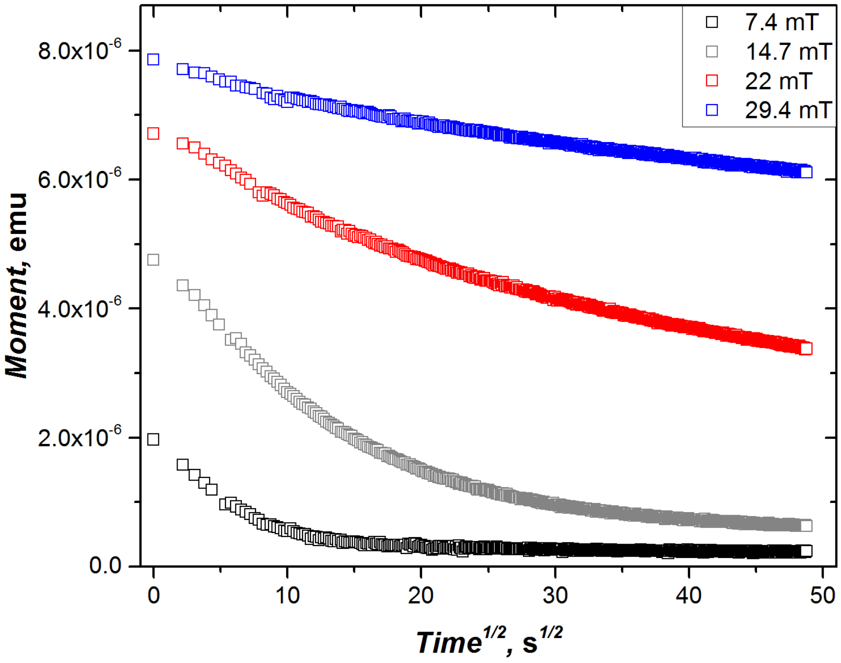

Figure S11. $DySc_2N@C_{80}$ single crystal magnetization decay curves at 1.8 K. The square-root time scaling is used in order to determine a specific relaxation regime with square-root time dependence (corresponds to straight line in this case).

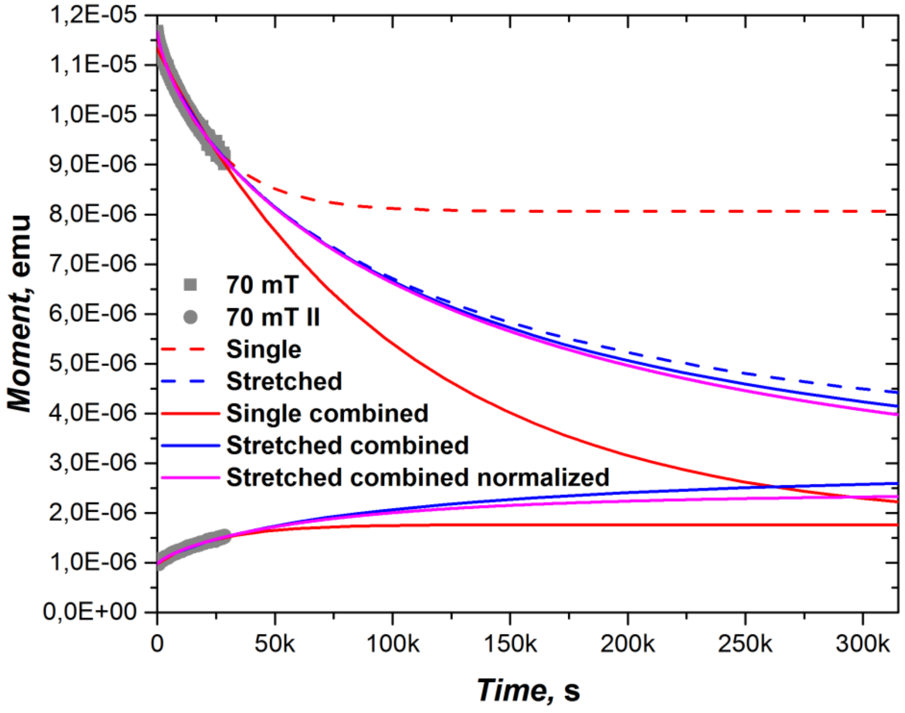

Figure S12. An example of a magnetization decay curve with a long relaxation time of ~1.2*10^5 seconds ($DySc_2N@C_{80}$, single crystal, 1.8 K, 70 mT).

Upon the increase of the relaxation time it becomes more and more time consuming to sample the complete relaxation curve. For the characteristic times of 10^5 seconds that would correspond to a 10 day experiment, which is already above the limit for a QD MPMS3 machine with unavoidable weekly liquid helium fillings (not mentioning the waste of the valuable SQUID time). The vital question then arises: how long is it necessary to measure the relaxation curve in order to obtain a reliable characteristic time?

The relaxation curve of $DySc_2N@C_{80}$ single crystal at 1.8 K, 70 mT is taken as an example. It has been measured for $2.8{\cdot}10^4$ seconds (~ 7 hours). Still in this case only a part of the decay curve was recorded (Figure S12). It is especially problematic to determine the equilibrium magnetisation for such incomplete curves. In order to increase the reliability of the fit, an additional "thermal" magnetisation curve can be recorded. With the magnetic field kept at the initial value (70 mT in the current example), one warms the sample above the blocking temperature and then cools it down to the initial temperature, where the measurement starts. The magnetisation is rising towards the same M_{eq} as for the decay curve. A combined fitting potentially yields more accurate results. In order to prove it, different fitting procedures are compared.

Single exponential fitting underestimates the characteristic relaxation time due to wrong value of M_{eq}, as it can be seen on Figure S12. Once a second "rising" experimental curve is added to the single exponential fitting ("Single combined"), the obtained relaxation time becomes comparable to ones from the fittings with stretched exponentials. The shape of the simulated curve, however, is still far from experimental. This

can be clearly seen at a closer look to the experimental curve (Figure S13). Only stretched exponentials that can closely follow the curvature of the magnetisation decay curve with a time-dependent relaxation rate. For the full experimental curve the different stretched exponential fittings provide similar simulated curves and the relaxation times. Thus, the most reliable average relaxation time of $(1.22\pm0.16)\cdot10^5$ seconds can estimated from 4 different fitting types: “Single combined”, “Stretched”, “Stretched combined”, “Stretched combined normalized” (Figure S14). The ratio between the duration of the measurement and the characteristic time is ~0.2. Further, the cases with smaller ratios were modelled.

The initial curves (both decay and magnetisation) were continuously shortened from the end in steps of $2.8\cdot10^3$ seconds (10% of the full measurement). The resulting set of curves was fitted with a range of methods, both with default optimization algorithm and with a "normalised" one. However, the results for "normalised" fitting were excluded if they are similar to the default one (Figure S14).

Following the Figure S14 one can see how the estimated relaxation time varies with the length on the experiment. In the case of single exponential fitting the estimated value changes continuously with the duration of the experiment. This is an additional argument against using this method.

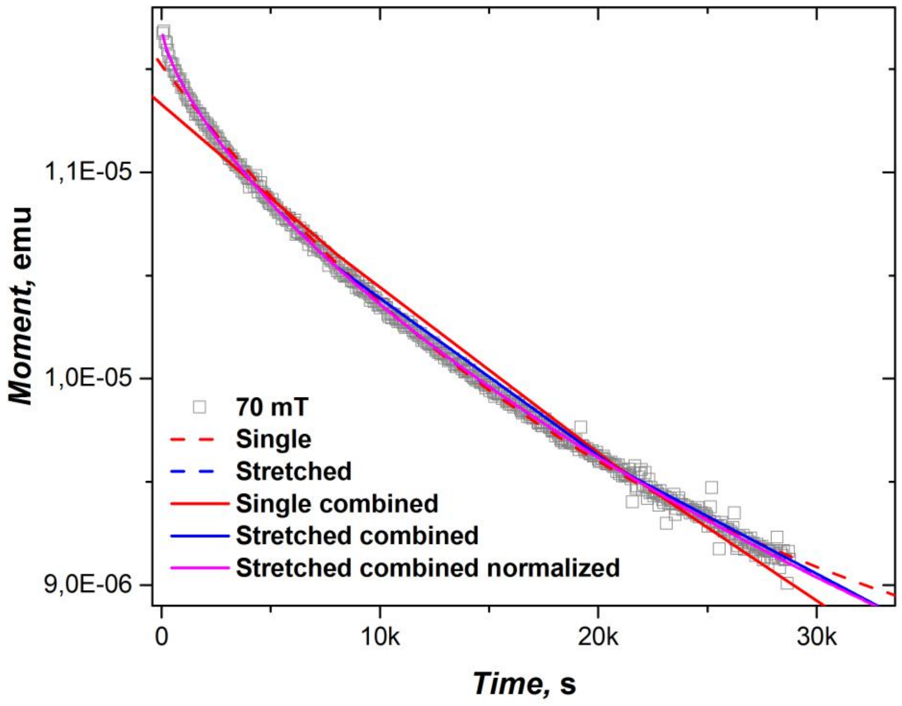

Figure S13. An example of a magnetization decay curve with a long relaxation time of $\sim1.2\cdot10^5$ seconds ($DySc_2N@C_{80}$, single crystal, 1.8 K, 70 mT).

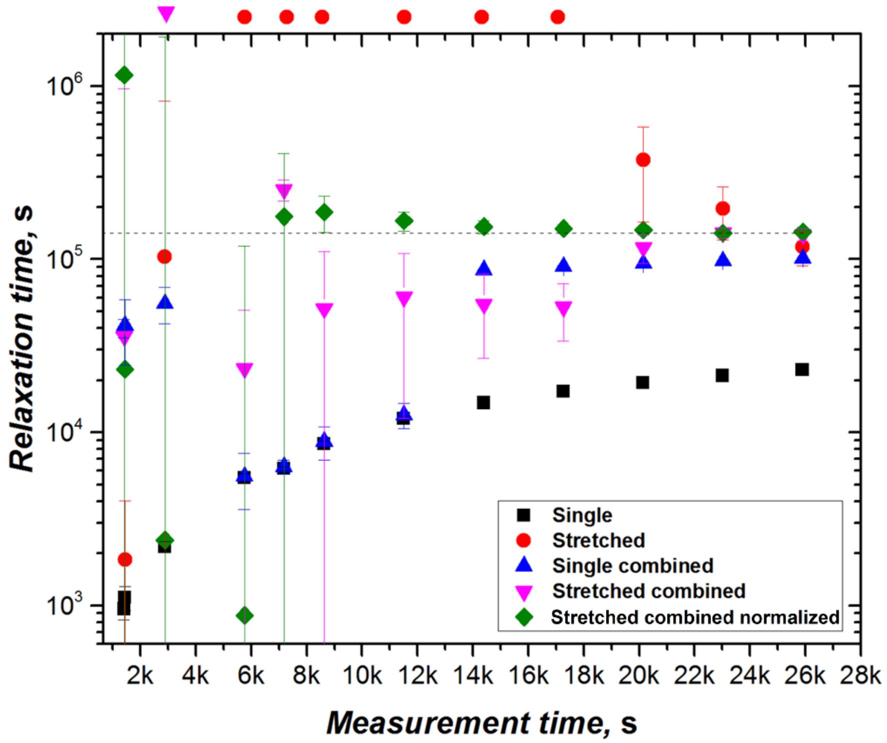

Figure S14. Relaxation times obtained from different pieces of the magnetization decay curve ($DySc_2N@C_{80}$, single crystal, 1.8 K, 70 mT). Points above the main frame represent strongly deviating times. The dashed line corresponds to the most reliable estimation of the relaxation time.

The value of the relaxation time obtained by using "Single combined" fitting stays nearly constant down to ~0.1 ratios (experiment time to relaxation time), then rapidly deviates and occasionally coincides with single exponential fitting.

"Stretched" fitting values start to deviate immediately upon shortening of the experimental curve and the deviation can reach several orders of magnitude. This happens due to unconstrained M_{eq} parameter that is difficult to estimate for a single decay curve.

"Stretched combined" method provides quite stable results, however, the standard deviation of the relaxation time value grows with shortening of the experiment. The reason is in the inappropriate weighting, which ignores the second "rising" magnetization curve as it has one order of magnitude smaller values than the decay curve (Figure S12).

The weight of the data points from both decay and magnetisation experimental curves have to be normalized in the same way as it was done above for the case of short relaxation times. Accordingly, the "Stretched combined normalized" fitting method shows more accurate results, which do not deviate down to experiment time to relaxation time ratio of ~0.05 for the current case. Thus, the reliable experiment

can't be more than 20 times shorter than the estimated relaxation time. The relaxation times determined for $DySc_2N@C_{80}$ samples in this work and listed in Tables S3-S7 were determined by this method.

Thus, we found that the stretched exponential is the optimal function for fitting the experimental DC relaxation curves. The following important notes can be pointed out:

- Normalization of the weights is required when the measured signal varies within more than one order of magnitude.
- For short relaxation times, the reliable values can be obtained down to ~100 s.
- For long relaxation times, when only a part of the decay curve is measured, an additional measurement of the "rising" magnetization curve is required for an accurate estimation of M_{eq} value. In this case two 30 h measurements will provide the reliable results for relaxation times up to ~10^6 seconds.

Magnetic relaxation times determined by DC magnetometry

Table S3. Relaxation times of powder $DySc_2N@C_{80}$ samples in the field of 0.2 T

	non-diluted powder			$DySc_2N@C_{80}$@MOF			$DySc_2N@C_{80}$@PS		
T	τ_m, s	st. dev., s	β	τ_m, s	st. dev., s	β	τ_m, s	st. dev., s	β
1.8	510687	9860	0.50	33661	138	0.59	60507	1224	0.55
2	70839	1089	0.55	19100	41	0.62	16647	459	0.71
2.2	20524	891	0.61	10102	24	0.64	7410	514	0.67
2.5	7589	76	0.66	3955	35	0.63	2470	47	0.75
3	1461	9	0.74	923	3	0.73	758	2	0.77
3.8	342.1	0.5	0.74	305.1	0.7	0.69	244.6	0.6	0.73
5	59.5	0.3	0.75	79.6	0.4	0.70	68.1	0.3	0.77

Standard deviation ("st. dev.") is the error of the fit

Table S4. Relaxation times of non-diluted powder $DySc_2N@C_{80}$ sample and of $DySc_2N@C_{80}$ in MOF at 1.8 K in different fields

	non-diluted powder			$DySc_2N@C_{80}$@MOF		
$\mu_0 H$, Oe	τ_m, s	st. dev., s	β	τ_m, s	st. dev., s	β
28				9970	38	0.36
78				19939	100	0.35
178				43589	370	0.34
278	105	5	0.36	68664	454	0.40
378	509	104	0.39	80212	525	0.46
478	12025	296	0.43	80035	631	0.50
578	31943	142	0.43	76347	631	0.52
678	73667	434	0.43	73293	622	0.53
978	284907	2851	0.44	59429	450	0.56
1478	485423	7242	0.48	44382	268	0.58
1978	510687	9860	0.50	33661	138	0.59
2478	347723	5819	0.54	25390	114	0.60
2978	214225	3976	0.59	19179	39	0.59
3478	143264	2602	0.62	14632	33	0.58
3978	95433	1271	0.64	11226	29	0.57
4478	69839	992	0.64	8615	23	0.55
4978	54502	748	0.63	6532	16	0.52
7478	23275	297	0.53	825	5	0.65

Table S5. Relaxation times of $DySc_2N@C_{80}$ diluted in MOF at 1.8 K in different fields

	Powder diluted in PS		
$\mu_0 H$, Oe	τ_m, s	st. dev., s	β
-22	68883	238	0.64
-12	68233	423	0.56
-7	37419	180	0.42
-2	299	2	0.56
0	92	1	0.56
3	386	2	0.45
8	41275	110	0.43
18	70115	619	0.61
28	79453	769	0.54
78	69705	600	0.47
178	86016	979	0.34
478	98129	1522	0.41
678	93390	1407	0.44
978	83446	1435	0.48
1478	75872	4287	0.40
1978	42473	2858	0.47
2478	16513	257	0.43
2978	9879	91	0.40
3478	11547	1529	0.45
3978	3989	48	0.38

Table S6. Relaxation times of diluted crystal at 1.8 K in different fields

$\mu_0 H$, Oe	τ_m, s	st. dev., s	β
14.5	368	38	0.55
51	3929	406	0.24
124	42271	5693	0.21
343	77289	36209	0.77
489	94019	16032	0.76
708	57635	40380	0.84
1073	45807	32313	0.91
1438	35436	10915	0.88
2168	28321	4246	0.69
2898	8396	445	0.58
3628	1824	205	0.47

Table S7. Relaxation times of non-diluted crystal at 1.8 K in different fields

$\mu_0 H$, Oe	τ_m, s	st. dev., s	β
125	179	4	0.54
198.5	3448	72	0.35
272	16794	7635	0.37
478	112074	31479	0.71
678	140502	5126	0.71
978	138888	18747	0.69
1478	125923	5556	0.68
1978	64174	4986	0.67
2978	19673	302	0.58
3978	4382	104	0.56

Magnetic relaxation time from AC magnetometry

Table S8. Relaxation times of non-diluted $DySc_2N@C_{80}$ powder measured at different temperatures in zero field

T, K	τ_m, s	st. dev., s	α
2	3.16	0.06	0.29
2.2	2.82	0.02	0.28
2.4	2.56	0.07	0.28
3	1.86	0.02	0.27
4	1.40	0.02	0.27
5	1.08	0.02	0.26
8	0.554	0.007	0.24
10	0.317	0.005	0.19
15	0.122	0.001	0.15
20	0.0574	0.0006	0.12
25	0.0348	0.0004	0.13
30	0.0220	0.0004	0.11
35	0.0156	0.0002	0.11
40	0.0110	0.0002	0.10
50	0.00596	0.00012	0.09
55	0.00451	0.00016	0.00
60	0.00261	0.00004	0.00
71	0.00177	0.00004	0.00
77	0.00114	0.00008	0.00
83	0.00085	0.00001	0.00
85	0.00074	0.00001	0.00
87	0.00062	0.00002	0.00

Fitting of zero-field magnetic relaxation times in the 2–5 K

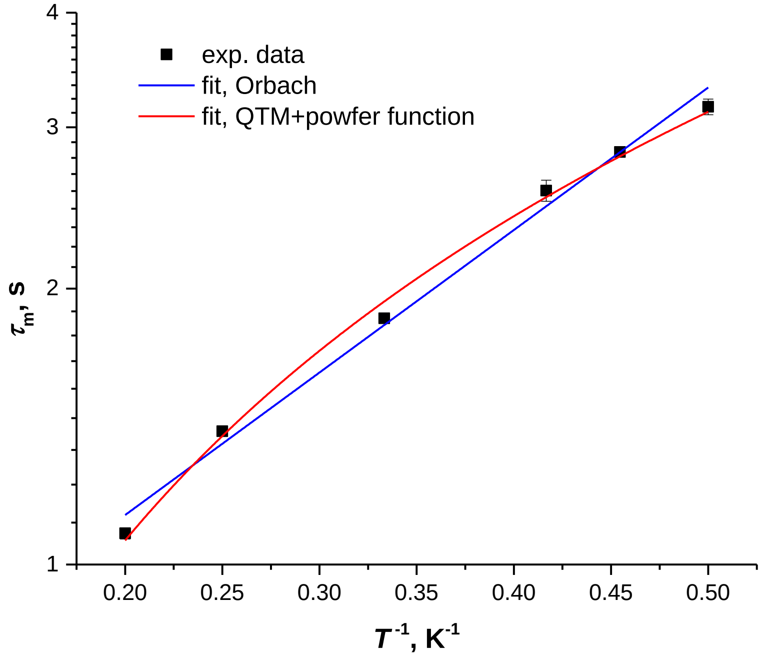

Figure S15. Relaxation times of powder $DySc_2N@C_{80}$ measured by AC magnetometry in zero DC field between 2 and 5 K. The lines are fits to the Orbach relaxation mechanism (blue) and a combination of QTM and a power function of temperature (red lines).

Orbach mechanism. *Fitting function*: $\tau_m^{-1}(T) = \tau_0^{-1}\exp(-U^{\mathrm{eff}}/T)$

Fitted parameters: τ_0^{-1} = 1.81 ± 0.09 s^{-1}, U^{eff} = 3.58 ± 0.13 K

Combination of QTM and a power function of temperature. Fitting function: $\tau_m^{-1}(T) = bT^n + \tau_{\mathrm{QTM}}^{-1}$,

Fitted parameters: b = 0.142 ± 0.006 s^{-1}K^{-n} and n = 1.18 ± 0.04, $\tau_{\mathrm{QTM}}^{-1} \approx 0$.

Fitting of magnetic relaxation times above 5 K

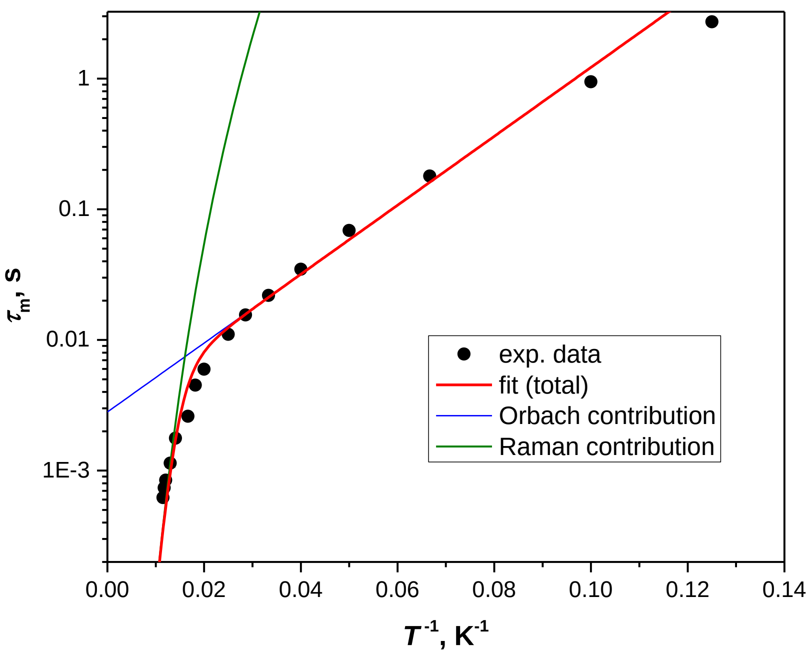

Figure S16. Relaxation times of magnetization of $DySc_2N@C_{80}$ (T>5 K, AC magnetometry), combination of zero-field measurements (T> 20 K) and in-field measurements (5 K < T < 20 K.) Fit with the combination of Orbach and Raman mechanisms, the exponent in the Raman process is set to n = 9.

Fitting function: $\tau_m^{-1}(T) = \tau_0^{-1}\exp(-U^{\mathrm{eff}}/T) + AT^9$

Fitted parameters: τ_0^{-1} = (2.8 ± 0.5)·10^{-3} s^{-1}, U^{eff} = 60.7 ± 3.0 K, A = (9.4±2.9)·10^{-15} s^{-1}K^{-9}

The function does not provide a good fit (deviations are significant). Besides, Raman process dominates at higher temperature and Orbach at lower temperature.

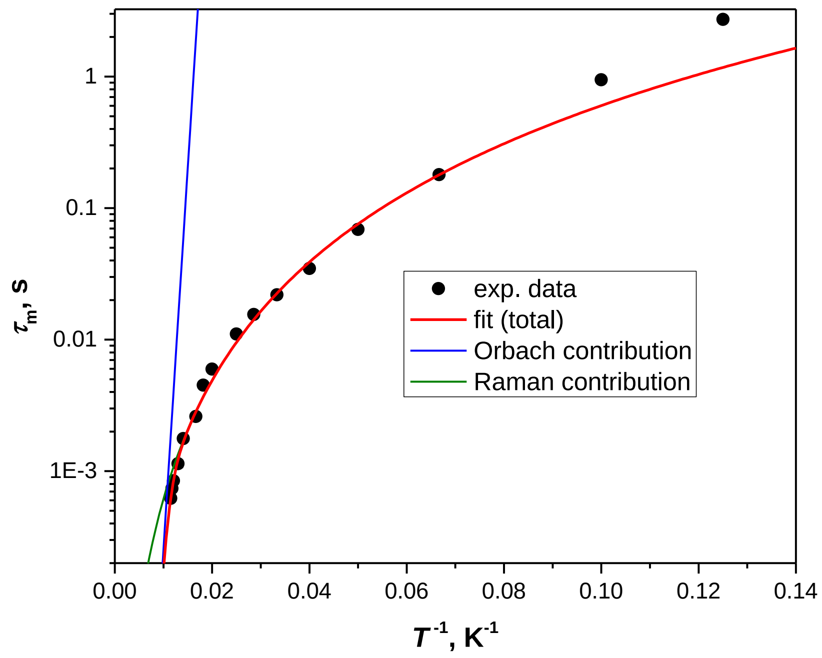

Figure S17. Relaxation times of magnetization of $DySc_2N@C_{80}$ (*T*>5 K, AC magnetometry), combination of zero-field measurements (*T*> 20 K) and in-field measurements (5 K < *T* < 20 K.) Fit with the combination of Orbach and Raman mechanisms similar to Fig. S16 above, but the exponent in the Raman process is also allowed to vary.

Fitting function: $\tau_m^{-1}(T) = \tau_0^{-1}\exp(-U^{\text{eff}}/T) + AT^n$

Fitted parameters: τ_0^{-1} = (3.9 ± 0.9)·10^{-10} s^{-1}, U^{eff} = 1338 ± 2009 K, n = 2.99±0.07, *A* = (1.7±0.2)·10^{-3} $s^{-1}K^{-n}$

As can be seen from the graph and from the errors in fitted parameters, the Orbach contribution in the fitted curve is negligible and hence non-reliable. Except for the two lowest-temperature points, the data are reasonably described by the power function of temperature with the exponent close to 3. This value is very different from the expected *n* = 9 for the Raman process in Kramers ion.

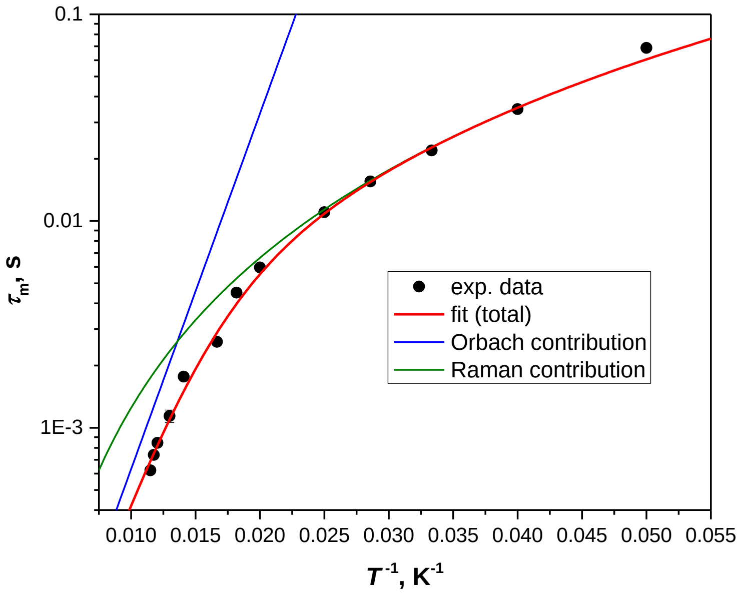

Figure S18. Relaxation times of magnetization of $DySc_2N@C_{80}$ (T>20 K, zero-field measurements, AC magnetometry). Fit with the combination of Orbach and Raman mechanisms, the exponent in the Raman process is also allowed to vary.

Fitting function: $\tau_m^{-1}(T) = \tau_0^{-1}\exp(-U^{\mathrm{eff}}/T) + AT^n$

Fitted parameters: τ_0^{-1} = (1.2 ± 0.7)·10^{-5} s^{-1}, U^{eff} = 396 ± 55 K, n = 2.41±0.18, A = (1.2±0.7)·10^{-2} $s^{-1}K^{-n}$

The data is reasonably described by the power function of temperature with the exponent close to 2.4 (which is very different form the expected n = 9 for the Raman process in Kramers ion) and the Orbach process at higher temperatures. Note dramatic changes in the fitted parameters compared to the Fig. S17 caused by a reduction of the data set by few low-temperature points. This points to the low stability of the fit and hence makes the results questionable.

Ab initio calculations for $DySc_2N@C_{80}$

Single point CASSCF/RASSI calculation was done for a DFT-optimized structure of $YSc_2N@C_{80}$ (Table S9) with substitution of Y by Dy. For Dy^{3+} ion the structure of high spin multiplet $^6H_{15/2}$ can be represented in eight low-lying Kramers doublets (Fig. S19). For ab initio modeling of the Dy multiplet structure we used Molcas 8.0 code.[15] The active space of the CASSCF calculations includes eleven active electrons and seven active orbitals (e.g. CAS (11,7)). All 21 sextet states and 108 quartets and only 100 doublets were included in the state-averaged CASSCF procedure and were further mixed by spin-orbit coupling in the RASSI procedure. VDZ quality atomic natural extended relativistic basis set (ANO-RCC) was employed. The single ion magnetic properties and ligand field parameters were calculated with use of SINGLE ANISO module (Table S10). The ab initio ligand field parameters were used to construct a model Zeeman Hamiltonian in |J,m_J>-basis (Clebsch-Gordan decomposition). Based on this Hamiltonian, the transition probabilities between different states were estimated using PHI program.[16]

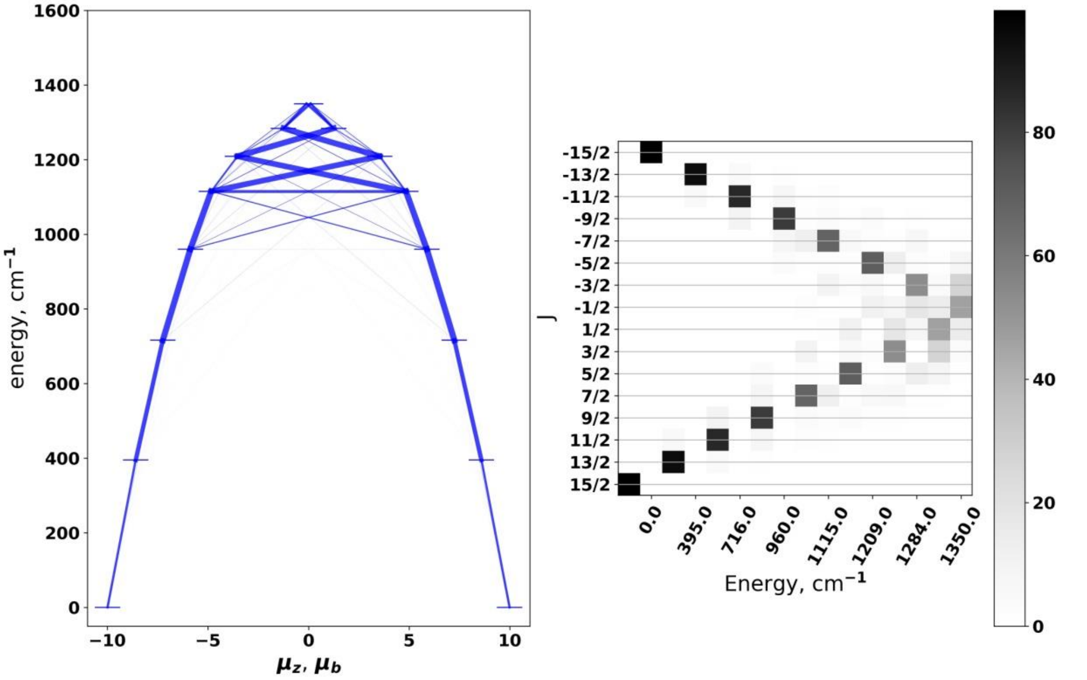

Figure S19 (Left) $^6H_{15/2}$ multiplets structures of Dy^{3+} ion as computed at the CASSCF/RASSI level of theory. The thickness of blue lines visualizes the transition probabilities between different |J,m_J> sates. (Right) Shows percentage composition of the states.

Table S9. Cartesian coordinated of $DySc_2N@C_{80}$ used in DFT calculations							
C	0.829454090	2.560678590	3.111986630	C	-2.236098430	3.261680700	1.148890300
C	0.447200690	1.418442890	3.894881040	C	-1.496561440	2.992618070	2.339094140
C	-0.971884740	1.097377700	3.984792450	C	-1.877075010	1.844734700	3.126644020
C	1.370563810	0.310090410	3.898552280	C	-0.125301030	3.367611710	2.379985230
C	2.081148090	2.617559140	2.390513330	C	-1.586574530	3.813267680	-0.010616480
C	3.692242640	1.260501460	1.152067420	C	0.533804020	3.910060330	-1.224228760
C	2.954991420	1.499310110	2.367273290	C	-0.184724030	4.093522210	-0.003206080
C	2.610723210	0.360405520	3.151860970	C	0.528703990	3.910295070	1.224031840
C	0.913375220	-1.074661100	3.896953210	C	-1.907947630	1.847684140	-3.143675760
C	-0.490070610	-1.413315350	3.902706350	C	-1.488424000	2.990178050	-2.362095810
C	-1.439784130	-0.307510270	3.992895930	C	-2.225979140	3.268345390	-1.178166430
C	-0.855872980	-2.547221430	3.099840900	C	-0.109569200	3.356032380	-2.388484970
C	1.875544590	-1.848111000	3.140561770	C	1.898406250	3.459851020	-1.214271800
C	2.233696230	-3.263109790	1.173506190	C	2.573995130	3.176258990	0.005876420
C	1.487504630	-2.981036600	2.359037330	C	1.893248100	3.447617270	1.228580430
C	0.110623120	-3.344288550	2.379435750	C	3.530691220	2.091947570	-0.006723400
C	4.077025740	-0.058386450	0.715413200	C	2.106744770	2.621175430	-2.371668810
C	3.613154590	-1.203238140	1.470741170	C	2.647424190	0.367418660	-3.120908630
C	2.927112520	-0.968645370	2.705325080	C	3.028415600	1.523910560	-2.363451880
C	3.272992940	-2.372826160	0.738926340	C	3.858445800	1.311199630	-1.187164100
C	-2.102730150	-2.622749280	2.363246400	C	-0.926700950	1.053432440	-3.825055650
C	-2.972063920	-1.503871500	2.323712910	C	1.400888920	0.305732830	-3.844114070
C	-2.606089090	-0.362013150	3.126252520	C	0.474608090	1.385830760	-3.795353510
C	-3.714557350	-1.269752680	1.122655780	C	0.859170850	2.561032500	-3.096285260
C	-1.903314620	-3.454020880	1.210261300	C	-0.828761230	-2.559033140	-3.115124120
C	-1.888488970	-3.432632390	-1.237387670	C	0.951156950	-1.059633180	-3.848669170
C	-2.576014860	-3.165578720	-0.019497560	C	-0.441932150	-1.385966280	-3.819123340
C	-3.522960940	-2.094048490	-0.038643870	C	-1.403326760	-0.306115830	-3.881075930
C	1.587140250	-3.793163210	0.002242240	C	2.235130330	-3.242747950	-1.156643830
C	0.186273070	-4.080967420	-0.005128060	C	1.909535700	-1.847201180	-3.120523420
C	-0.532816800	-3.893404980	1.215992270	C	1.505535860	-2.979092070	-2.352434270
C	-0.526367900	-3.891355950	-1.233234540	C	0.127176200	-3.357902570	-2.396912810
C	-4.035879630	0.057847170	0.677923300	C	4.226113220	-0.050644810	-0.738803500
C	-3.603683840	1.199177980	1.416037180	C	2.973235210	-0.974341460	-2.674865700
C	-2.895004210	0.959154020	2.642908910	C	3.720014530	-1.215365680	-1.467931630
C	-3.276398450	2.374638830	0.694569580	C	3.285902670	-2.356599430	-0.710592490
C	-4.077694890	0.060683060	-0.768351720	N	0.157145810	0.052096020	0.140706680
C	-3.041692160	1.000000890	-2.807131750	Sc	2.011914240	0.136376090	-0.544437850
C	-3.683360140	1.225695510	-1.519443000	Sc	-0.292424520	0.063975270	2.073791510
C	-3.278438960	2.372949180	-0.751013830	Dy	-1.477471100	-0.060399780	-1.266602130
C	-2.094065350	-2.602596000	-2.407892330				
C	-3.060598020	-1.533995960	-2.462076190				
C	-3.765772000	-1.291081880	-1.223922780				
C	-2.735508560	-0.367338270	-3.287727990				

Table S10. Ab initio crystal field parameters in Steven's notation		
k	q	B_{kq}
2	-2	-9.56584E-001
2	-1	2.19113E+000
2	0	-7.68758E+000
2	1	-3.80138E-001
2	2	-8.49367E-001
4	-4	1.32174E-003
4	-3	3.44572E-005
4	-2	-8.96999E-004
4	-1	5.07593E-003
4	0	-4.42360E-003
4	1	-9.60419E-004
4	2	1.56363E-003
4	3	-9.74794E-003
4	4	-2.31873E-004
6	-6	9.04360E-006
6	-5	7.77853E-005
6	-4	1.20841E-004
6	-3	-3.23471E-005
6	-2	1.23290E-005
6	-1	-4.11278E-004
6	0	3.84396E-006
6	1	5.78083E-005
6	2	1.32594E-004
6	3	-3.48031E-004
6	4	-6.14676E-005
6	5	8.91142E-004
6	6	6.01888E-005

Simulation of magnetic hysteresis curves from spin dynamics

System settings. Let's consider the 2-level system with the energy gap Δ and coupling term ($\Omega \neq 0$):

$$\mathcal{H}_{\mathcal{S}} = \hbar \frac{\Delta}{2} \hat{\sigma}_z + \hbar \frac{\Omega}{2} (\hat{\sigma}_+ + \hat{\sigma}_-) \quad \text{(S3)}$$

Such limited Hamiltonian is often used in resonance fluorescence studies.[17-19] Having supplied this system Hamiltonian into the GKSL equation, one will get a phenomenological master equation:

$$\dot{\rho} = -i \frac{\Delta}{2} [\hat{\sigma}_z, \rho] - i \frac{\Omega}{2} [\hat{\sigma}_+ + \hat{\sigma}_-, \rho] + \mathcal{L}(\gamma_e \mathcal{A}) \rho. \quad \text{(S4)}$$

By setting γ_e and the operator $\mathcal{A}$, we will allow one state to "relax" onto another with relaxation rate γ_e. We can set the environment coupling operator $\mathcal{A}$ to be proportional to a raising ($\hat{\sigma}_+$) or lowering ($\hat{\sigma}_-$) operator depending on the relaxation direction. For example, if the lowering is chosen and $\mathcal{L}(\hat{\sigma}_-)\rho$, the master equation would be read as following:

$$\begin{aligned} \dot{\rho} = -i \frac{\Delta}{2} [\hat{\sigma}_z, \rho] - i \frac{\Omega}{2} [\hat{\sigma}_+ + \hat{\sigma}_-, \rho] + \\ + \frac{\gamma_e}{2} (2\hat{\sigma}_- \rho \hat{\sigma}_+ - \hat{\sigma}_- \hat{\sigma}_+ \rho - \rho \hat{\sigma}_- \hat{\sigma}_+). \end{aligned} \quad \text{(S5)}$$

Any generic two-level quantum system has the Hilbert space isomorphic to a spin (or pseudo spin)-half system and thus can be considered as one. The Hamiltonian Eq. S3 and the master equations Eq. S4/S5 together produce a suitable theoretical framework for magentodynamcis modeling. In that case the states energy split is linear with field $\Delta = \Delta(H) \sim H$ and the parameter Ω is a non-zero probability to tunnel between these states. The expectation value of $\langle \hat{\sigma}_z(t) \rangle$ is magnetization by definition ($\langle | \uparrow\rangle\langle\uparrow | (t) - |\downarrow\rangle\langle\downarrow | (t) \rangle \equiv M(t)$). Having that said, one can conduct a magnetization measurement experiment for observation period δ and in field H. To make this experiment possible, however, we have to introduce a self-evident field sweeping axioms.

Field sweep axioms

We assume that during the field sweep the system response is non-adiabatic locally, *e.i.* between two consequent field points, and adiabatic globally.

Let us prepare (or magnetize) the system Eq. S3 into the up-state by using field $H = h$. Next, we will sweep the field backwards from h to -h following the given rules:

- from point to point the field is changed instantly, so is the Hamiltonian components ($\Delta(H_i)$ to $\Delta(H_{i+1})$);
- at each point system stays for an observation period δ, during which magnetic moment evolved unperturbed according to equation of motion;
- magnetic moment determination (observation) collapses the states on a "median-state", so that initial wavefunction operating on the new Hamiltonian (step: $i + 1$) is defined as $|\Psi\rangle(0) = \langle u^i(t) \rangle_\delta(0) | \uparrow\rangle + \langle d^i(t) \rangle_\delta(0) | \downarrow\rangle$, where $d^i(t)$ and $u^i(t)$ expectation values for the operators $|\downarrow\rangle\langle\downarrow|$ and $|\uparrow\rangle\langle\uparrow|$ respectively, during observation time δ on the Hamiltonian at step i;

Here is how this propagation algorithm works. Figure S20 shows for forward (in blue) and backward (in red) propagation of the systems described by Eq. S4. The dependence of time-averaged magnetization as a function of H or simply $\mathcal{M}(H) \equiv \langle M(t) \rangle_\delta$ is derived for different values of Ω in the range of 0.1-0.4 $[\frac{h}{\delta}]$, while the maximum energy difference is in the highest field $\Delta(h) = 1[\frac{h}{\delta}]$. Thus, it appears that if $\Omega/\Delta(h) <$ < 0.1 on the field sweep from h to $-h$ the state would not switch.

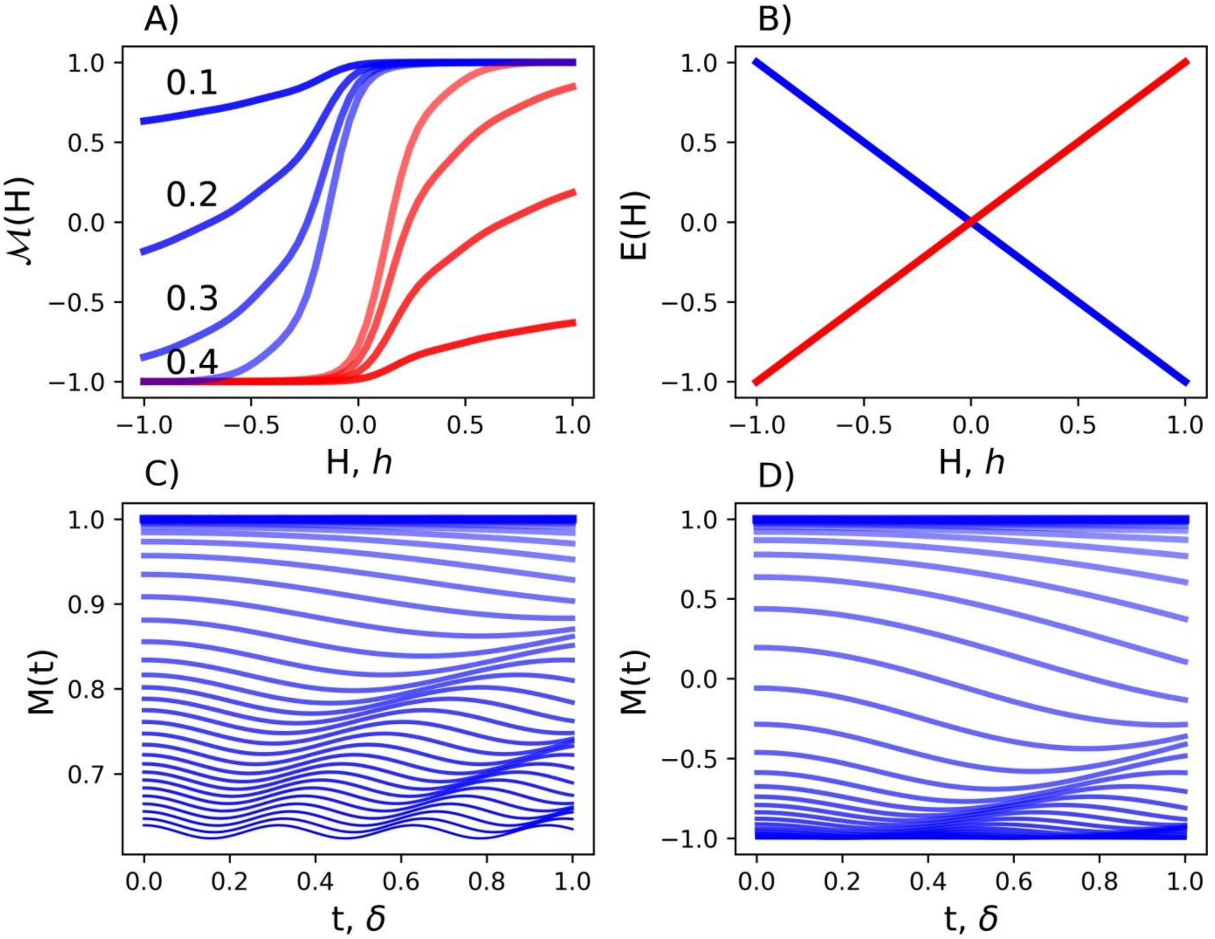

Figure S20 (A) $\mathcal{M}(H) \equiv \langle M(t) \rangle_\delta$as function of the field H[h], each point along the sweep observed for δ with $\Omega \cdot \delta$ ratio 0.1, 0.2, 0.3 and 0.4. (C) and (D) magnetization dynamic in each point for the sweep for $\Omega \cdot \delta$ equal 0.1 and 0.4 respectively. (B) State energy as a function of field.

Same field sweep algorithm is used in hysteresis modeling in the main text.

Prove of correct thermodynamic limit and connection to LLG theory.

In the limit $\gamma_e >> \Omega$ for any H and $T > 0$ the master equation, so eq. S5 is read as:

$$\dot{\rho} \simeq -i\frac{\Delta}{2}[\hat{\sigma}_z, \rho] + \mathcal{L}(\sqrt{\gamma_e}(d\hat{\sigma}_- + u\hat{\sigma}_+))\rho \qquad \text{(S6)}$$

where $d = e^{\frac{-\beta}{2}\varepsilon^+}/Z$ and $u = e^{\frac{-\beta}{2}\varepsilon^-}/Z$. Using the cyclic properties of the trace, the equation of motion for expectation values of $\hat{\sigma}_u =$ and $\hat{\sigma}_d$ reads as follows:

$$\begin{aligned} \frac{d}{dt}\langle\hat{\sigma}_u\rangle &= \gamma_e(-d^2\langle\hat{\sigma}_u\rangle + u^2\langle\hat{\sigma}_d\rangle) \\ \frac{d}{dt}\langle\hat{\sigma}_d\rangle &= \gamma_e(+d^2\langle\hat{\sigma}_u\rangle - u^2\langle\hat{\sigma}_d\rangle) \end{aligned} \qquad \text{(S7)}$$

This set of equations is nothing but the semi-classical optical Bloch-equation identical to the ones which can be derived from Landau-Lifshits-Gilbert model.[20, 21] This identity justifies the physical meaning of γ_e and confirms that approach operates within physically relevant roam. Furthermore, recalling that $\langle\hat{\sigma}_d\rangle + \langle\hat{\sigma}_u\rangle = 1$ which leads to a trivial solution:

$$\begin{aligned} \langle\hat{\sigma}_u\rangle &= \frac{u^2 + d^2 e^{-\gamma_e(u^2+d^2)t}}{u^2 + d^2} \xrightarrow{[t\to\infty]} \frac{u^2}{u^2 + d^2} = \\ &= \frac{e^{-\beta\varepsilon^-}}{e^{-\beta\varepsilon^-} + e^{-\beta\varepsilon^+}} = P_u(T) \end{aligned} \qquad \text{(S8)}$$

here $P_u(T)$ is Boltzmann probability to find system in the up-state at temperature T.

References

1. S. Yang, L. Zhang, W. Zhang and L. Dunsch, *Chem.-Eur. J.*, 2010, **16**, 12398-12405.
2. D. S. Krylov, F. Liu, S. M. Avdoshenko, L. Spree, B. Weise, A. Waske, A. U. B. Wolter, B. Büchner and A. A. Popov, *Chem. Commun.* , 2017, **53**, 7901-7904.
3. A. G. Orpen, L. Brammer, F. H. Allen, O. Kennard, D. G. Watson and R. Taylor, *J. Chem. Soc., Dalton Trans.*, 1989, S1-S83.
4. J. Zhang, W. Yi, Z. Chen and X. Zhou, *Dalton Trans.*, 2013, **42**, 5826-5831.
5. S. Anfang, K. Harms, F. Weller, O. Borgmeier, H. Lueken, H. Schilder and K. Dehnicke, *Z. Anorg. Allg. Chem.*, 1998, **624**, 159-166.
6. K. L. M. Harriman, J. L. Brosmer, L. Ungur, P. L. Diaconescu and M. Murugesu, *J. Am. Chem. Soc.*, 2017.
7. L. Zhao, S. Xue and J. Tang, *Inorg. Chem.*, 2012, **51**, 5994-5996.
8. P. Zhang, L. Zhang, C. Wang, S. Xue, S.-Y. Lin and J. Tang, *J. Am. Chem. Soc.*, 2014, **136**, 4484-4487.
9. D. M. McCluskey, T. N. Smith, P. K. Madasu, C. E. Coumbe, M. A. Mackey, P. A. Fulmer, J. H. Wynne, S. Stevenson and J. P. Phillips, *ACS Appl. Mat. Interfaces* 2009, **1**, 882-887.
10. R. Westerström, J. Dreiser, C. Piamonteze, M. Muntwiler, S. Weyeneth, K. Krämer, S.-X. Liu, S. Decurtins, A. Popov, S. Yang, L. Dunsch and T. Greber, *arXiv:cond-mat/0604365v1*, 2013, 1310.5040.
11. I. Koponen, *Journal of Non-Crystalline Solids* 1995, **189**, 154-160
12. J. C. Phillips, *Rep. Prog. Phys.* , 1996, **59** 1133–1207.
13. L. Thomas, A. Caneschi and B. Barbara, *Physical Review Letters*, 1999, **83**, 2398-2401.
14. A. Caneschi, D. Gatteschi, C. Sangregorio, R. Sessoli, L. Sorace, A. Cornia, M. A. Novak, C. Paulsen and W. Wernsdorfer, *J. Magn. Magn. Mater.*, 1999, **200**, 182-201.
15. F. Aquilante, J. Autschbach, R. K. Carlson, L. F. Chibotaru, M. G. Delcey, L. De Vico, I. Fdez. Galván, N. Ferré, L. M. Frutos, L. Gagliardi, M. Garavelli, A. Giussani, C. E. Hoyer, G. Li Manni, H. Lischka, D. Ma, P. Å. Malmqvist, T. Müller, A. Nenov, M. Olivucci, T. B. Pedersen, D. Peng, F. Plasser, B. Pritchard, M. Reiher, I. Rivalta, I. Schapiro, J. Segarra-Martí, M. Stenrup, D. G. Truhlar, L. Ungur, A. Valentini, S. Vancoillie, V. Veryazov, V. P. Vysotskiy, O. Weingart, F. Zapata and R. Lindh, *J. Comput. Chem.*, 2016, **37**, 506-541.
16. N. F. Chilton, R. P. Anderson, L. D. Turner, A. Soncini and K. S. Murray, *J. Comput. Chem.*, 2013, **34**, 1164-1175.
17. H.-P. Breuer and F. Petruccione, *The Theory of Open Quantum Systems*, Oxford University Press, Oxford, New York, 2002.
18. E. T. Jaynes and F. W. Cummings, *Proc. IEEE*, 1963, **51**, 89-109.
19. F. W. Cummings, *Phys. Rev.*, 1965, **140**, A1051-A1056.
20. F. Bloch, *Phys. Rev.*, 1956, **102**, 104-135.
21. G. V. Skrotskiĭ, *Sov. Phys. Uspekhi*, 1984, **27**, 977.
